# Hydrogeophysical characterization and recharge potential of three Wadi basins along the Red Sea Margin, Northeastern Desert, Egypt

**DOI:** 10.1038/s41598-026-37853-9

**Published:** 2026-02-27

**Authors:** Mahmoud Hussein, Sultan A.S. Araffa, Mahmoud Ahmed Abbas, Mahmoud S. Sharkawy

**Affiliations:** 1 Geology Department, Faculty of Science, Assiut Branch, Al-Azhar University, 71524 Assiut, Egypt; 2 National Research Institute of Astronomy and Geophysics (NRIAG), Helwan, Cairo, 11421 Egypt; 3 Department of Earth, Environmental and Resources Sciences (DiSTAR), University of Naples Federico II, Naples, 80126 Italy; 4 Geology Department, Faculty of Science, Qena University, Qena, 83523 Egypt; 5 Department of Petroleum Geology, Faculty of Petroleum and Mining Sciences, Matrouh University, Marsa Matrouh 51511, Matrouh, Egypt

**Keywords:** Northeastern desert, Egypt, Digital elevation model, Morphometric analysis, Structural control, Vertical electrical sounding (VES), Basement morphology, Middle miocene aquifer, Archie’s equation, Managed aquifer recharge, Environmental sciences, Hydrology, Solid Earth sciences

## Abstract

In the arid Red Sea margin setting, episodic Wadi runoff is a primary mechanism for groundwater recharge; its effectiveness depends on surface morphology, structural fabric, and subsurface architecture. This study assessed groundwater potential and recharge dynamics in Wadi Ramliya, Wadi Umm Alda, and Wadi Hamad in Northeastern Egypt by integrating morphometric, geophysical, hydrochemical, and meteorological datasets to prioritize sites for managed aquifer recharge and reconnaissance drilling. High-resolution Digital Elevation models (DEMs) and multi-azimuth hillshades were used to map topographic zoning and structural trends, while morphometric analysis quantified drainage metrics. Flood-event hazard mapping was developed by integrating the Suez rain gauge station as a reference, satellite-based rainfall from the CHIRPS dataset, and data on DEM, slope, drainage density, land-use/land-cover, and road distance. Twenty-eight Vertical Electrical Soundings (VES) were used to constrain a six-layer geoelectric model, and Archie-based transforms (ρw = 2.85 Ω.m) were applied to the estimated formation factors and porosities. Water quality was classified using hydrochemical data from the JICA-5 borehole, while land magnetic surveys, utilizing Total magnetic intensity (TMI), Reduced to the Pole (RTP), and analytic signal filters, delineated basement structural highs and depocenters to guide structural targeting. DEM/hillshade analysis delineated four topographic zones (17.5–1,292 m) and showed dominant NW–SE and NE–SW trends that control drainage orientation. Morphometric indices highlight contrasting basin behavior; Wadi Hamad (A = 46.8 km²) exhibits the highest drainage density (D_d_ = 1.79 km km⁻²), relief ratio (R_r_=31.3), and bifurcation ratio (R_b_=3.0), consistent with steep, structurally guided, flash-prone headwaters. By contrast, Wadi Ramliya (A = 452.6 km²) and Wadi Umm Alda (A = 377.4 km²) act primarily as transit and depositional basins. The VES interpretation yielded three principal curve types (QH, HK, and QQ), with QH dominating the dataset. It resolves a hydraulically significant Middle Miocene calcareous-sandstone aquifer at 77–122 m (with resistivity of 12–23 Ω.m). Archie-derived formation factors (F = 4.2–8.2) imply porosities of 35–49% (mean ≈ 40.6%); resistivity-based saturation remained low (illustrative S_w_= 5.6%). JICA-5 chemistry classified the deeper layer (layer-6) as slightly brackish (TDS = 2,447 mg L⁻¹). Magnetic depth estimates and analytic-signal mapping identified shallow structural highs and deeper depocenters that correlated with depositional fans and VES targets. Flood-hazard mapping identified low-slope fan toes and coastal plain cells as high-priority recharge locations, which coincide with favorable geophysical signatures. Integrated data indicate that managed recharge pilots at alluvial fan toes and targeted reconnaissance drilling at lineament intersections are the highest-priority field actions; these should be accompanied by downhole logging, pumping tests, water-quality monitoring, and sediment control measures.

## Introduction

**Fig. 1:**
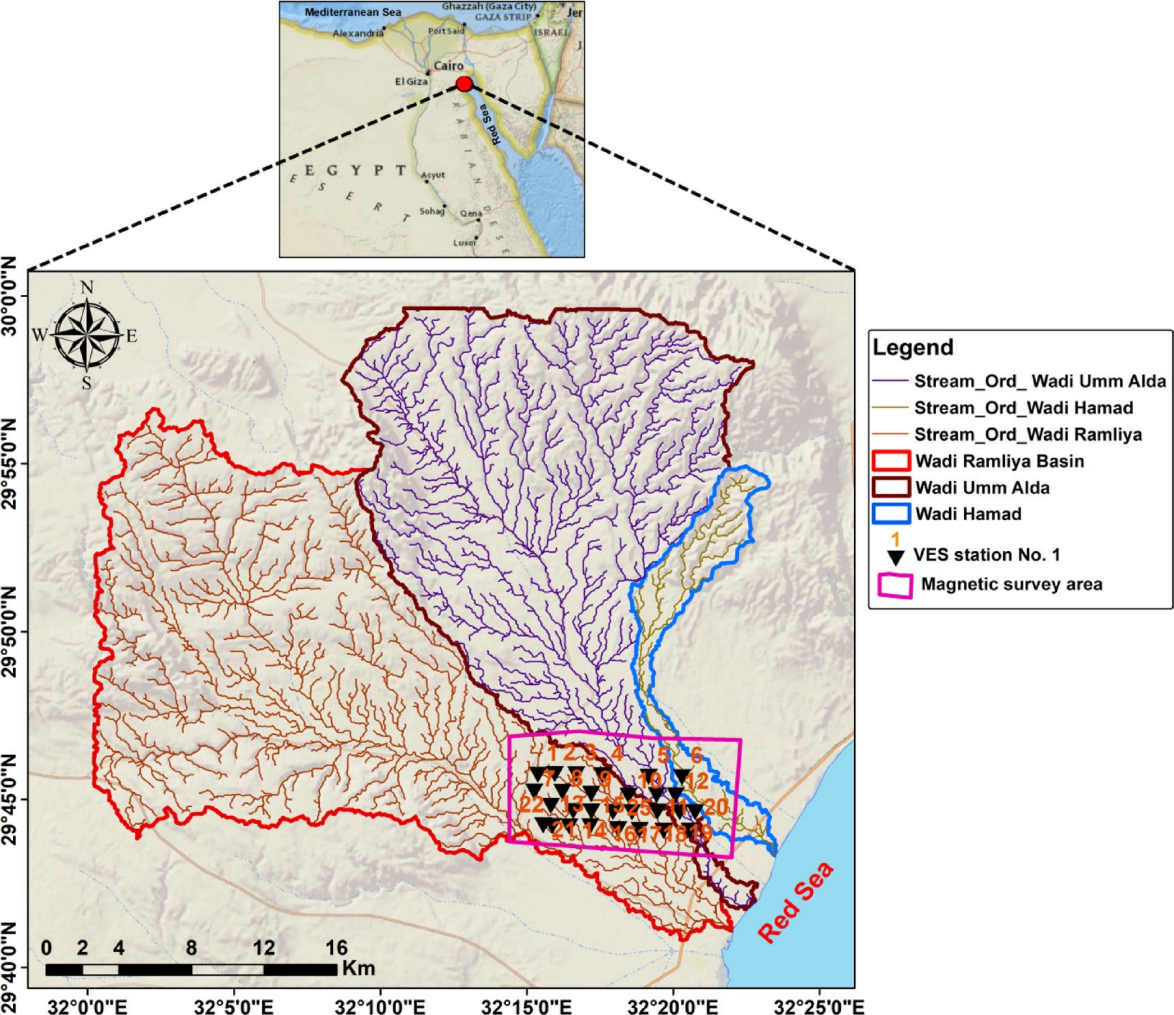
Location map of the study area.

Water resources in arid and semi-arid areas are closely connected to the landscape system, subsurface architecture, and active tectonics. In the northeastern desert of Egypt, seasonal wadis supply occasional surface runoff that recharges alluvial aquifers and, wherever structurally favorable, feeds deeper fracture-hosted aquifers, a process documented for Red Sea margin basins^1^. The area records a transition from stable shelf deposition to rift-related deformation (Upper Cretaceous to Quaternary), reflecting the long-term evolution of the Red Sea–Gulf of Suez rift system (Fig. 1)^2,3^. This stratigraphic succession and its subsequent reactivation by rifting produced a structural fabric dominated by NW–SE and NE–SW trends that control drainage orientation, relief, and subsurface permeability^3,4^.

Landscape metrics and geophysical imaging provide interdependent tools for mapping structural control: high-resolution Digital Elevation Models (DEMs) and multi-azimuth hillshades delineate topographic zoning and expose the spatial distribution of ridges, basins, and lineaments that act as watershed divides and preferred flow paths, while morphometric parameters (drainage density, bifurcation ratio, form factor, ruggedness) quantify basin form and hydrologic response^5^. Magnetic field data and the electrical resistivity method, using Vertical Electrical Sounding (VES), provide constraints on basement geometry, sediment thickness, and aquifer continuity. Integrating these datasets reduces ambiguity, resolves the ambiguities of single-source datasets, and yields a robust, site-scale framework for targeting recharge zones and drilling locations^2,4,6^.

To achieve the study goals, complementary methods were combined. Remote sensing (high-resolution DEMs and multi-azimuth hillshades) was used to map surface morphology, drainage networks, and lineaments over large areas; however, it cannot directly image buried units or fluid content, and its results depend on data resolution and processing choices^2,4,6^. Land magnetic surveys were applied to image basement morphology, large-scale structural trends, and depth variations of magnetic sources; however, magnetic anomalies are non-unique and insensitive to non-magnetic sedimentary contrasts, requiring careful filtering and modeling. Geoelectrical soundings (1-D Vertical Electrical Sounding (VES)) were used to detect resistivity contrasts related to lithology, clay content, and pore-fluid salinity, and were calibrated with borehole chemistry (Archie transforms) for petrophysical estimates, so far these methods are point-constrained, show depth resolution tradeoffs, and depend on assumed formation water resistivity and empirical parameters^7^. Flood-event and recharge potential were assessed by combining DEM-derived morphometrics (slope, drainage density), land use/cover, and rainfall records (Suez gauge/CHIRPS satellite rainfall) following established multi-criteria procedures^8,9^. By integrating surface mapping, magnetic constraints, and resistivity/borehole data, single-method ambiguities were reduced, and targeting of recharge zones and reconnaissance drilling was improved.

Accordingly, this study employed an integrated approach in three adjacent drainage basins: Wadi Ramliya, Wadi Umm Alda, and Wadi Hamad. These basins span a gradient in scale and relief (A = 452.6, 377.4, and 46.8 km², respectively) and thus provide an ideal natural laboratory for linking morphometry, structure, and hydrogeology. The datasets employed in this study include a high-resolution DEM, multi-azimuth hillshades, mapped lineaments, stream order and drainage density extractions, 28 VES stations with 1-D inversions, magnetic gridded data (Total magnetic intensity (TMI), Reduced to the Pole (RTP), and analytic signal data), and chemical analyses from a calibration borehole JICA-5. This study aims to evaluate flood events and recharge potential by combining rainfall records (gauge/CHIRPS rainfall) with DEM-derived morphometric data. These results are synthesized with magnetic and geoelectrical evidence to characterize aquifer layers and prioritize sites for managed recharge and exploratory drilling.

By integrating gauge/CHIRPS rainfall, magnetic, and geoelectrical data with VES and borehole measurements, this study maps drainage and structural controls, characterizes shallow and deep Middle Miocene aquifer layers (continuity and petrophysical properties), evaluates flood and recharge potential, and prioritizes sites for managed aquifer recharge and exploratory drilling.

## Geology of the study area

**Fig. 2:**
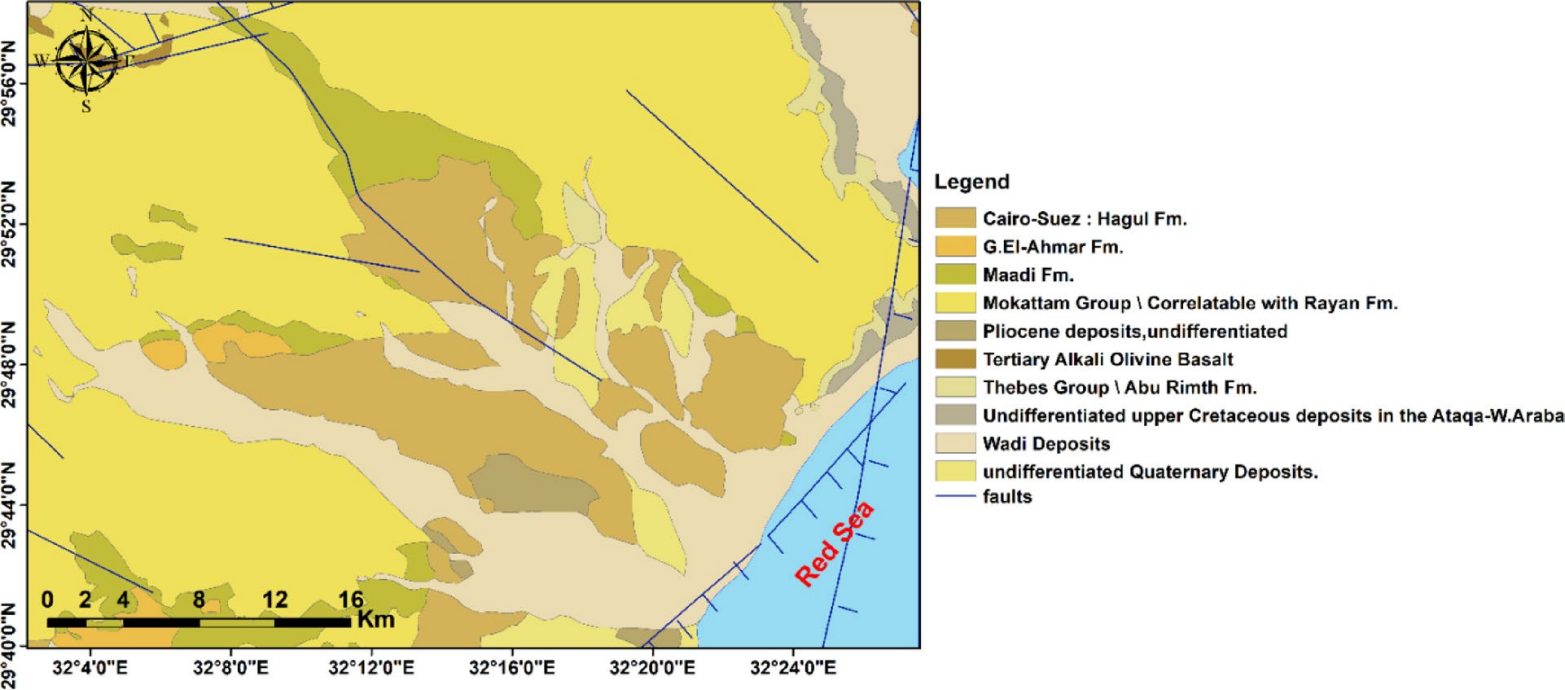
Geologic map of the study area.

The geological succession of the northeastern desert in Egypt ranges from the Upper Cretaceous to the Quaternary (Fig. 2). The oldest exposed rocks are undifferentiated Upper Cretaceous siliciclastic–carbonate deposits of the Ataqa–Wadi Araba region, which accumulated in shallow marine shelf settings^10^. These are overlain by extensive Eocene carbonate sequences, including the Thebes Group, Mokattam Group, and Hagul Formation, which are dominated by chalky limestones, marls, and nummulitic facies, indicating deposition in warm, shallow epicontinental seas^11^. The Oligocene succession is represented by the Gabal El-Ahmar and Maadi formations, which consist of ferruginous sandstones, clays, and lignitic beds deposited under fluvio-deltaic to marginal marine conditions. These formations are regarded as pre-rift accumulations preceding the Red Sea–Gulf of Suez rift^2^. During the Miocene–Pliocene, tectonic activity associated with rifting triggered basaltic volcanism, as reflected in the occurrence of Tertiary alkali olivine basalt.

Meanwhile, contemporaneous Pliocene sediments document post-rift subsidence and marine incursions^12^. Finally, the Quaternary cover comprises unconsolidated alluvial and wadi deposits together with coastal sediments, reflecting erosion, climatic oscillations, and ongoing tectonic activity that shaped the modern landscape of the area^13^. Collectively, the stratigraphic and structural framework illustrates the transition from stable shelf deposition in the Cretaceous–Eocene to Oligocene pre-rift fluvial systems, and then to rift-related volcanism and sedimentation during the Neogene–Quaternary.

## Methods

In this study, remote sensing data, geoelectrical resistivity measurements, and magnetic field data were utilized. Hillshade maps were generated from the DEM using eight illumination azimuths (0°, 45°, 90°, 135°, 180°, 225°, 270°, and 315°) at a solar altitude of 45°. Lineaments were extracted, and their orientations were analyzed using rose-diagram analysis and circular statistics. The vertical electrical soundings were used to determine the thickness and apparent resistivities of the lithological layers. Multi-layer models are manually reduced to a smaller number of layers and then iterated until the best fit is achieved between the calculated and observed curves. The analytical method was utilized in the present investigation to analyze VES measurements, determine layer resistivities and thicknesses, and infer aquifer characteristics. Magnetic data were prepared as a reduced-to-pole (RTP) grid for the study area, followed by standard processing and interpretation procedures. The initial steps included grid reprojection and datum registration, followed by diurnal and IGRF corrections, and the separation of regional and residual fields. Subsequent analyses applied reduction-to-pole, analytic-signal computation, and enhanced structural trends for mapping and interpretation.

### Remote sensing data

The Shuttle Radar Topography Mission (SRTM) 1-arc-second (30 m) digital elevation model (DEM) was used to generate hillshade and shaded-relief products for automated lineaments extraction and morphometric analysis. SRTM tiles covering the study area (e.g., SRTM1N29E32V3; acquisition date: 11 February 2000; release date: 23 September 2014) were obtained from the U.S. Geological Survey Earth Resources Observation and Science (EROS) Center via the LTA/Earth Explorer service^14^.

**Fig. 3:**
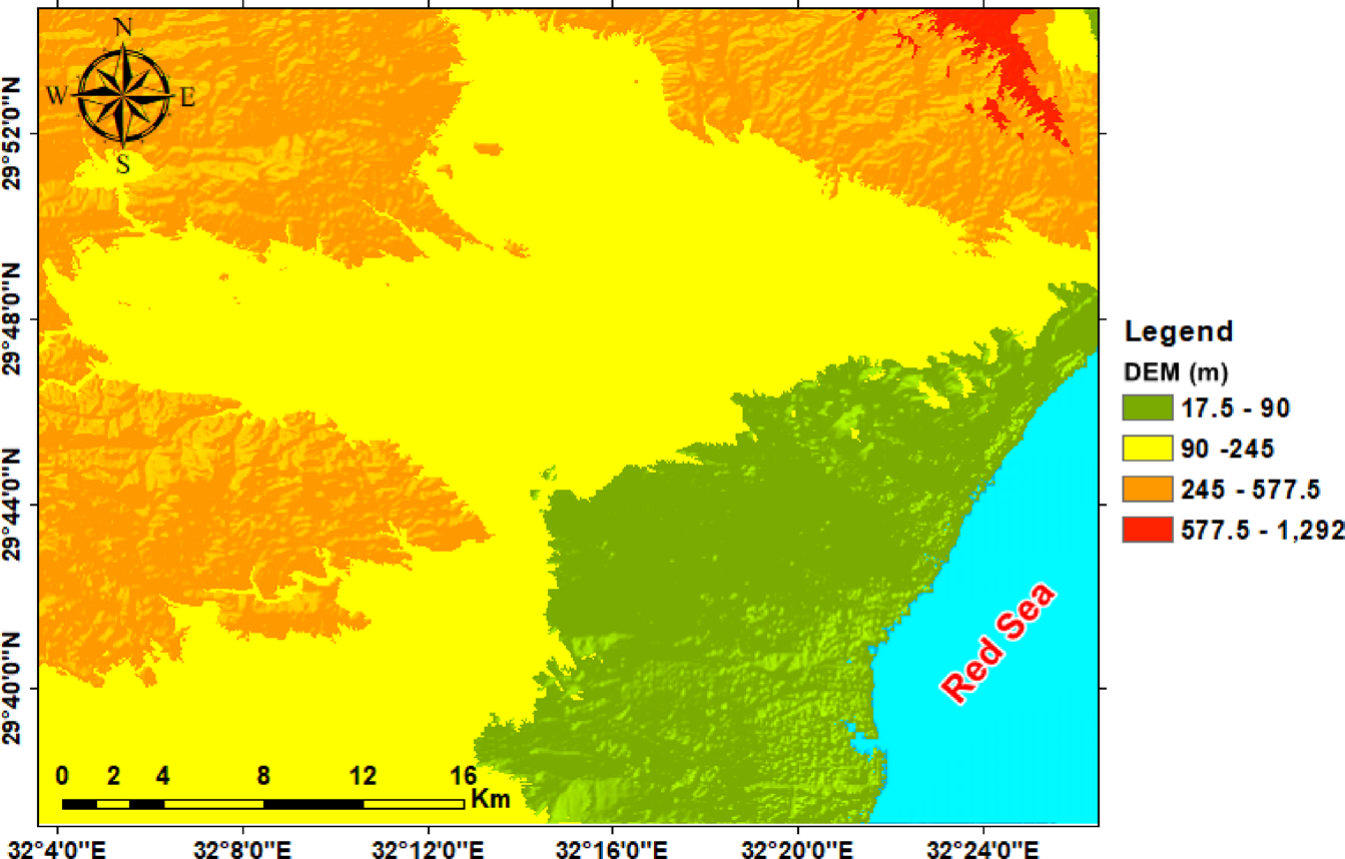
Digital elevation model showing the major topographic features of the study area.

**Fig. 4:**
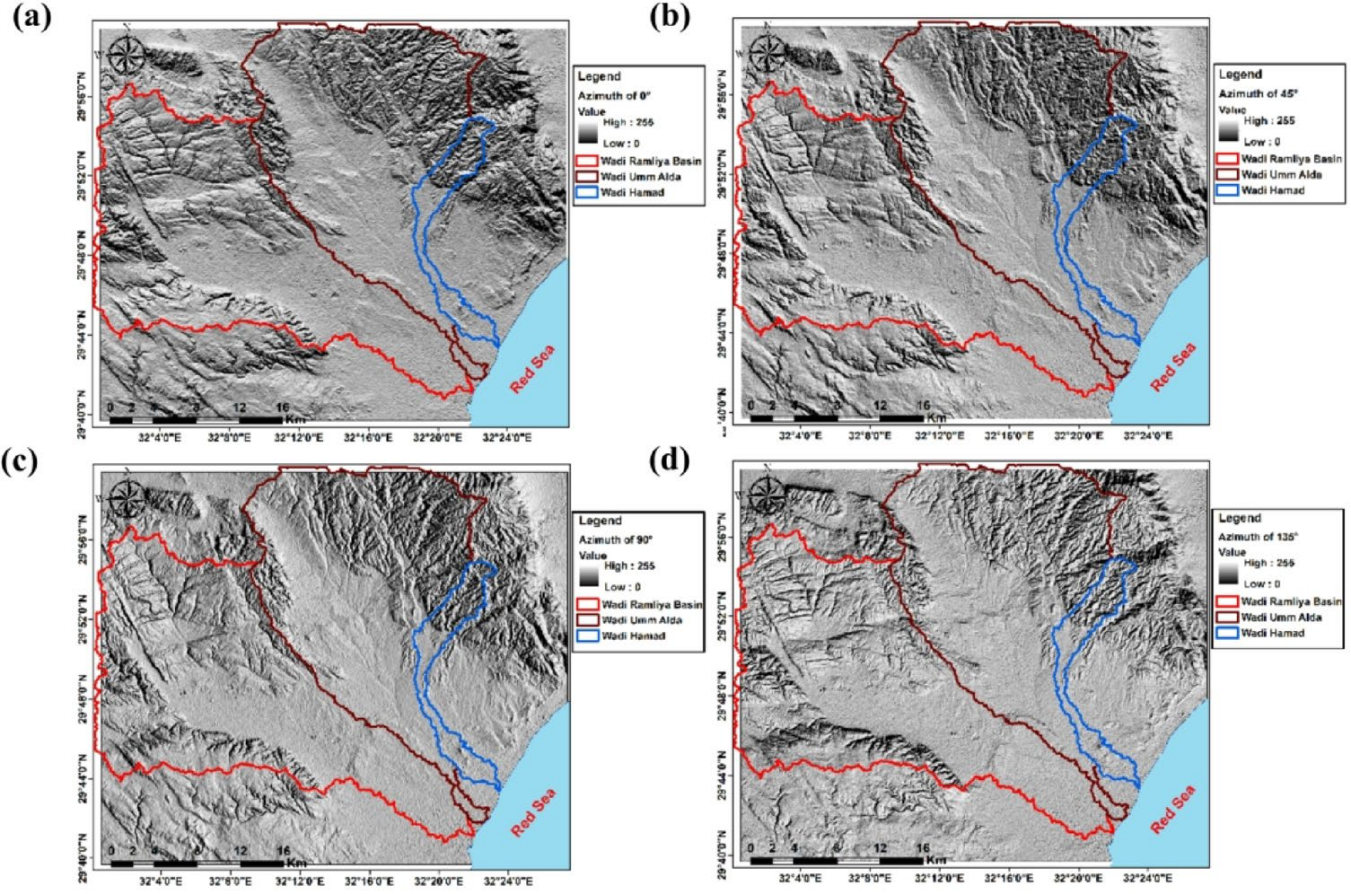
Hillshade maps of the study area were generated from the DEM using sun azimuths of 0° (**a**), 45° (**b**), 90° (**c**), and 135° (**d**) (solar altitude 45°).

The digital elevation model of the observed region reveals elevation values ranging from 17.5 to 1,292 m above sea level, with 4 predominant topographic zones (Fig. 3). The low-lying coastal plain (17.5–90 m) borders the Red Sea margin and forms a flat to gently sloping region, whereas wadis debouch, creating important recharge and discharge areas. Inland, the intermediate lowlands (90–245 m) occupy much of the central study area, forming broad basins and interfluves that collect runoff from adjacent uplands. Highland terrain (245–577.5 m) is primarily found in the western and northwestern parts and is characterized by rugged ridges and dissected slopes that serve as watershed divides. Mountainous terrain (577.5–1,292 m) is concentrated in the northeastern corner, where steep ridges and enhanced catchments act as primary recharge zones and sources of structurally controlled drainage. Overall, the DEM highlights the assessment contrast between rugged highlands and gently sloping coastal plains, showing ridges as watershed boundaries and the intervening basins as natural drainage collectors. The hillshade maps generated at azimuths of 0°, 45°, 90°, and 135° emphasize the main topographic and structural features of the study area (Fig. 4). Different sun angles highlight ridges and valleys of various orientations: zero° and 90° carry out east–west and north–south trends, whilst 45° and 135° display oblique NE–SW and NW–SE lineaments. These trends correspond to tectonic structures leading to the drainage of Ramalia, Umm Alda, and Hamad in the direction of the Red Sea. Therefore, hillshading complements the DEM by means of clarifying drainage and structural control of basin morphology.

### Meteorological data and flash-flood inventory

**Fig. 5:**
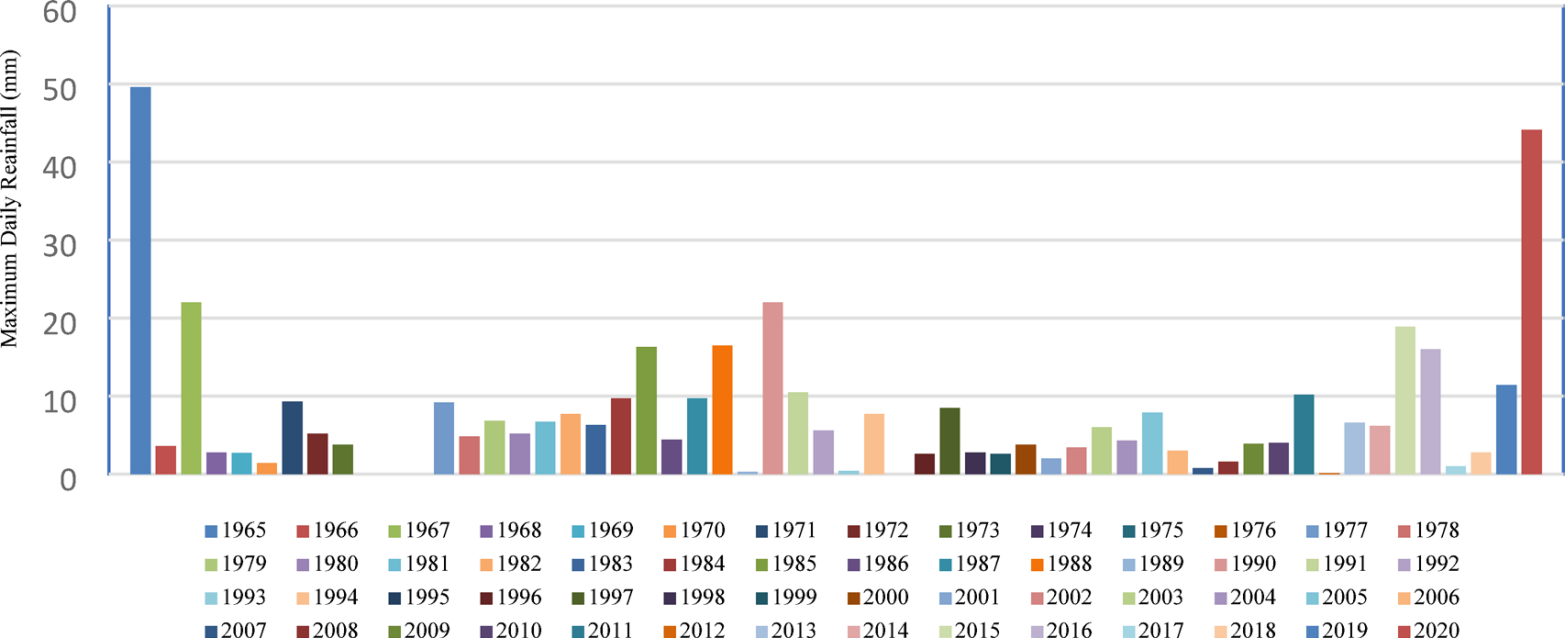
Maximum daily historical rainfall (1965–2020) from the Suez rain-gauge station.

Historical rainfall data for the study area were collected from the Suez Rain Gauge station in Suez City, located at 29°57′49″ N, 32°33′46″ E, which is the nearest ground meteorological station, approximately 40 km away. Due to the absence of rain gauges directly in the study area, we rely on Annual Maximum Daily Rainfall data for the period 1965–2020, as published by Refaey et al. ^8^, as shown in Fig. 5. To provide spatial rainfall coverage and fill gaps, we also utilized the CHIRPS satellite rainfall product website (https://chrsdata.eng.uci.edu/*)* for the same period.

**Fig. 6:**
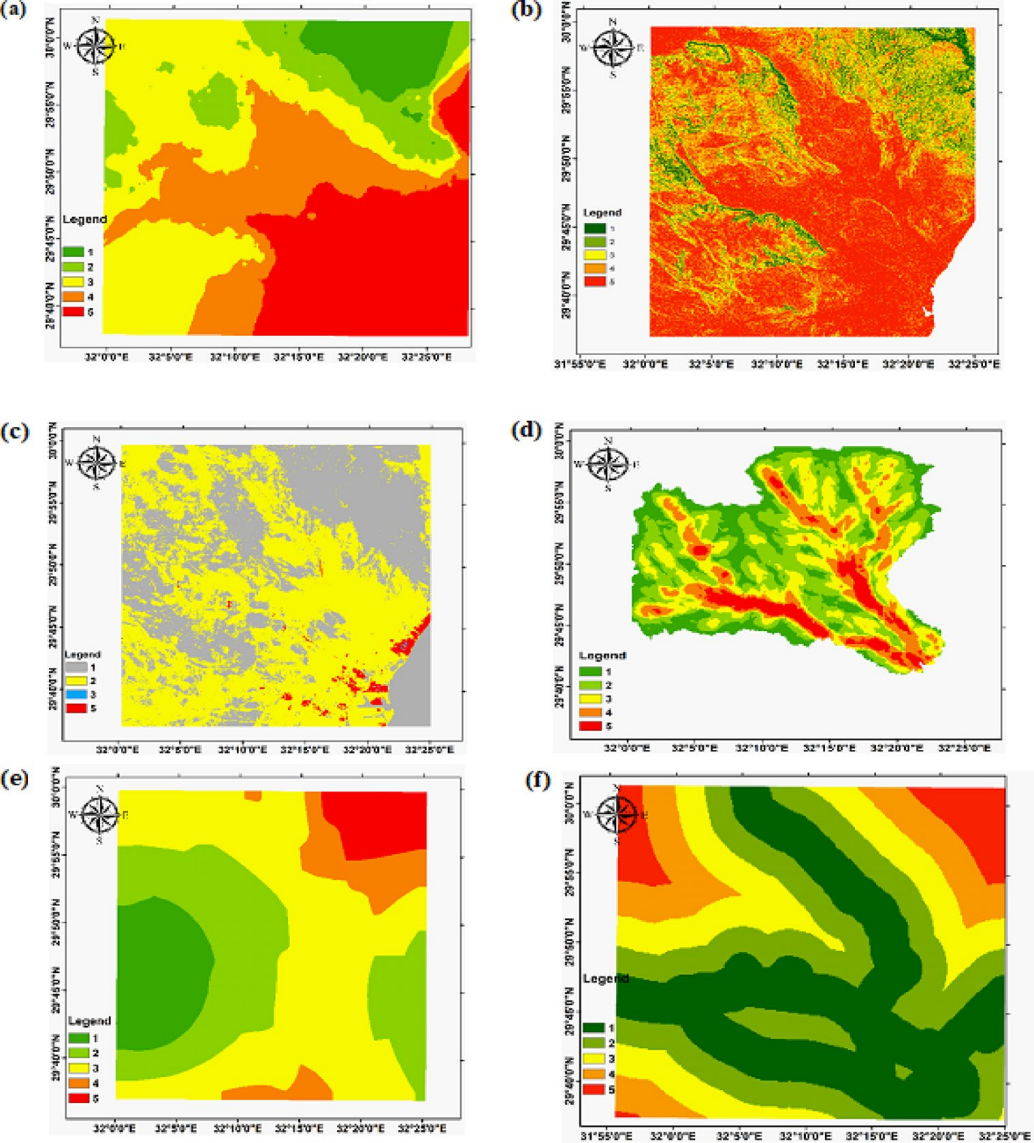
Flood event criteria reclassified: (**a**) Elevation; (**b**) Slope; (**c**) Land-use/cover; (**d**) Drainage Density; (**e**) Rainfall; (**e**) Distance from Road.

For developing the flood event unit, there is no consensus on the criteria that should be included^9^. The Digital Elevation Model (DEM) and other important parameters for flood event mapping were analyzed to assess topography, drainage patterns, and potential flood-prone areas. Flood-producing aspects, such as drainage density, digital elevation model, land use/land cover, rainfall, distance to roads, and slope, were also considered in assessing flood vulnerability. Input units were reclassified into categorical hazard classes (Very low to Very high); reclassified inputs are shown in Fig. 6, and the scoring scheme is summarized in Table 1.

**Table 1:** Common High-Weight reclassified factors include: Rainfall, drainage Density, land Use/Cover, Elevation, and Slope.

Event level	Hazard class	Rainfall (mm/day)	Drainage density (km·km⁻²)	Land-use/Land-cover (score)	Elevation (m)	Slope (degree)
1	Very low	≤ 27	≤ 2.00	≤ 2.0	≥ 624.35	≤ 6.0
2	Low	27.1–28.0	2.1–3.60	2.1–5.0	468.27–624.35	6.1–14.7
3	Medium	28.1–29.0	3.61–5.38	5.1–7.0	311.00–468.26	14.8–25.7
4	High	29.1–30.0	5.39–7.12	7.1–9.0	156.00–311.00	25.8–39.4
5	Very high	≥ 30	≥ 7.12	≥ 9.0	≤ 155.00	≥ 39.4

### Geoelectric data

**Fig. 7:**
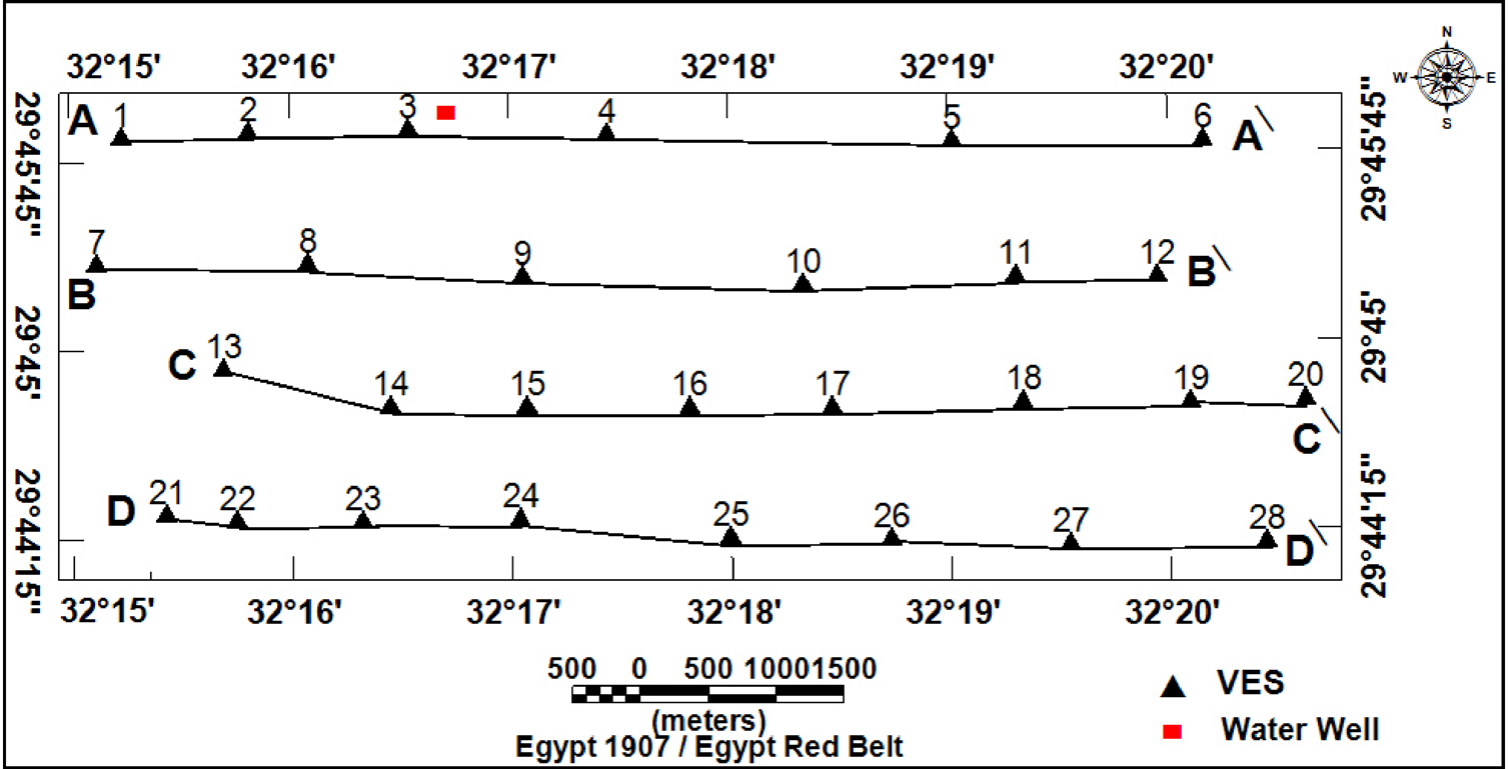
Locations of VES, borehole, and the four geoelectrical profiles (A–A′, B–B′, C–C′, and D–D′) within the study area.

**Fig. 8:**
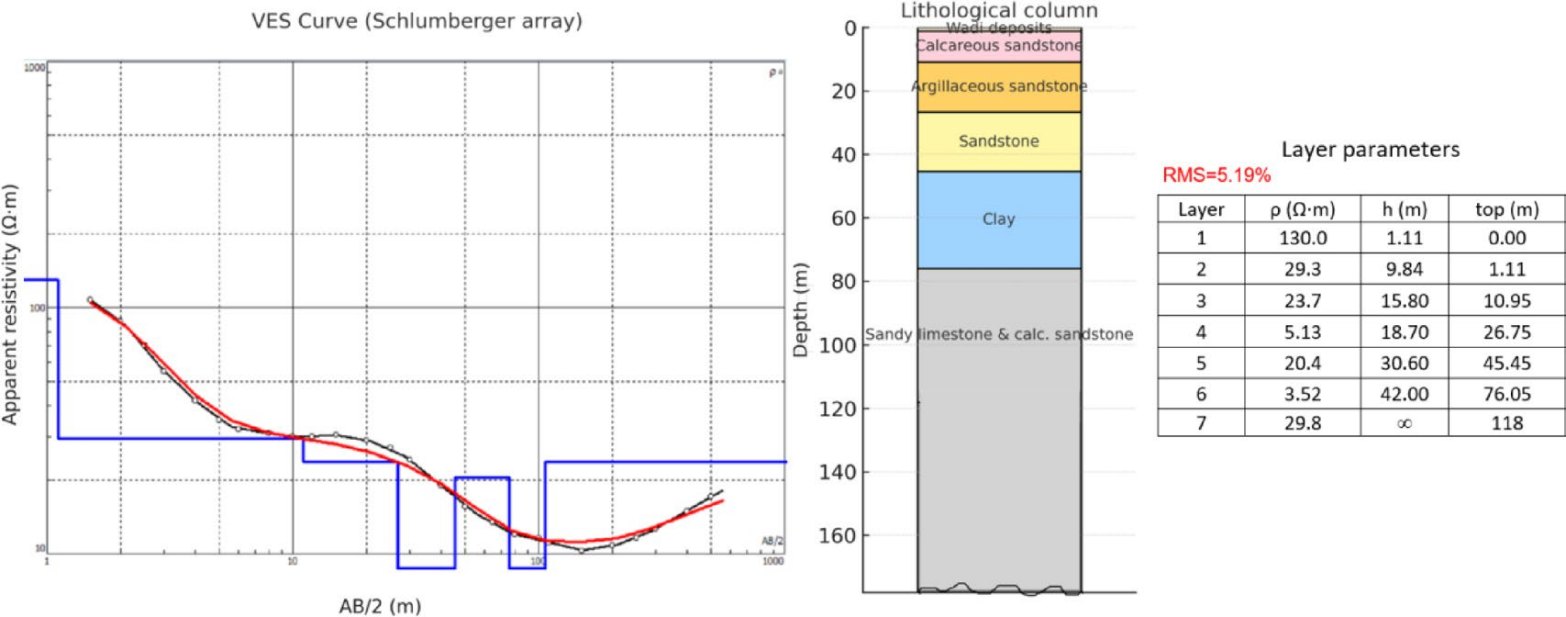
Resistivity models from VES No. 3 correlated with the geological log from borehole JICA-5.

Twenty-eight VES stations using a Schlumberger array with AB/2 ranging from 1.5 to 500 m were carried out. The VES station locations were distributed over an area extending 9230 m in length and 3460 m in width (Fig. 1). To delineate subsurface geoelectrical layers and the distribution of water-bearing layers, four west-east cross-sections were constructed across the study area (Fig. 7). A borehole drilled by the Egyptian Geological Survey^15^(JICA-5) is located 3 km north of VES No.3, which was used to correlate borehole geological information with geoelectric parameters derived from resistivity data (Fig. 8).

**Fig. 9:**
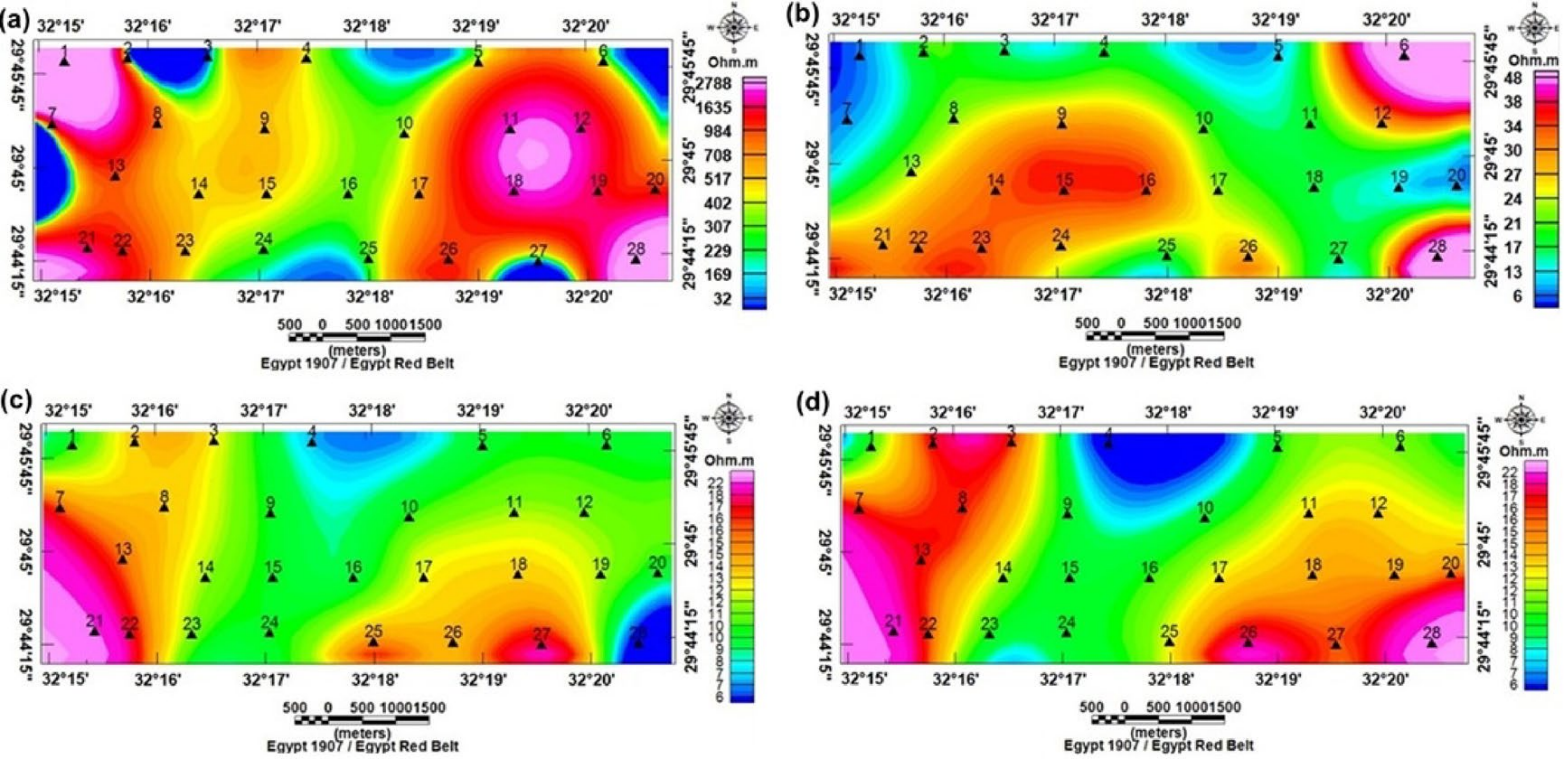
Iso-apparent resistivity maps at AB: (**a**) 3 m, (**b**) 60 m, (**c**) 600 m, and (**d**) 1000 m, showing the locations of VES stations.

Plan view apparent resistivity maps at shallow, intermediate, and deep AB/2 ranges exposed systematic lateral variations. At near-surface AB/2 (3 m; effective depth ~ 0.5–1 m), very high resistivity pockets (~ 1,800 Ω.m) had been observed in parts of the SE and western parts (especially at VES 28, 26, 20, 19, 18, 12, and 11) (Fig. 9a), consistent with coarse, dry gravels. At intermediate depths (AB/2 = 60 m; effective depth ~ 10–15 m) resistivities in central and southwestern zones increased moderately (24–48 Ω.m) whereas northern parts remained low (6–20 Ω.m) (Fig. 9b). At the largest AB/2 used (600–1,000 m; effective depths ≳ 100 m) most stations displayed commonly low resistivity values (~ 6–22 Ω.m) (Fig. 9c and d), except for scattered western and SE stations that retained relatively higher values. This depth is reliant on patterns suggested by shallow resistive coarse deposits overlying more conductive saturated or clay-rich horizons at depth.

### Hydrogeophysics estimates

Hydrogeophysics is implemented to map subsurface layers, estimate petrophysical properties, and assess groundwater quality across the study area. Twenty-eight VES were carried out to produce a consistent 6 geoelectric layers, which were then integrated with DEM, lineament, and lithologic information to identify aquifer architecture and recharge pathways. Beyond mapping the geometry of permeable wadi-fan sediments, the geoelectrical survey was critical for identifying hydraulic barriers (clay horizons) and characterizing the regional Middle Miocene calcareous sandstone aquifer. Combined with petrophysical analysis, these results provide insight into the spatial continuity and aquifer quality of the targeted groundwater layers.

The most common necessities for estimating porosity are readily available in this area, enabling the application of Archie’s Eq. 1^16^. The formation factors (F) of the Middle Miocene aquifer at selected locations are calculated according to the equation below:

1 $$F{\text{ }} = {\text{ }}{\rho _o}/{\text{ }}{\rho _w}$$

where 𝜌_o_ is the VES inferred resistivity of the water saturated formation, and 𝜌_𝑤_ is the resistivity of formation water (measured from the JICA-5, 𝜌w = 2.85 Ω.m). Porosity is then obtained from:

2 $$\varphi {\text{ }} = {\text{ }}{\left( {a/{\text{ }}F} \right)^{1/m}}$$

with a = 1, m = 2 (lithology coefficients appropriate to calcareous sandstone), which for the average formation factor F = 6.1 gave:

3 $$\varphi \% {\text{ }} = \sqrt {} {\text{ }}1/{\text{ }}6.1{\text{ }} \times {\text{ }}100 = 40.49\%$$

Water saturation is estimated from the Archie-derived form by means of rearranging the resistivity relation (Rt=R_w_FS_w_^−n^), yielding:

4 $$\;{S_w} = {(F{\rho _{w{\text{ }}/}}{\rho _t}_)^{1/n}}$$

### Magnetic data

**Fig. 10:**
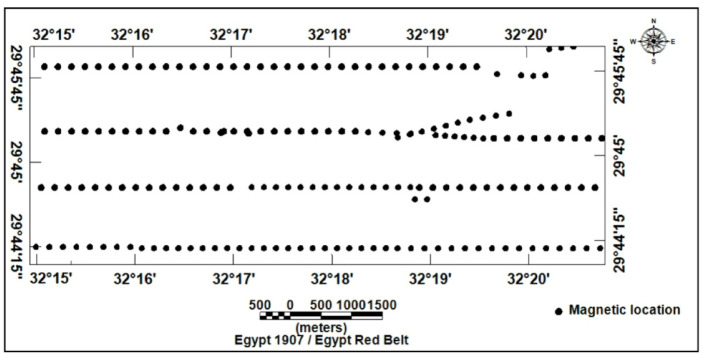
Location map of magnetic survey stations.

**Fig. 11:**
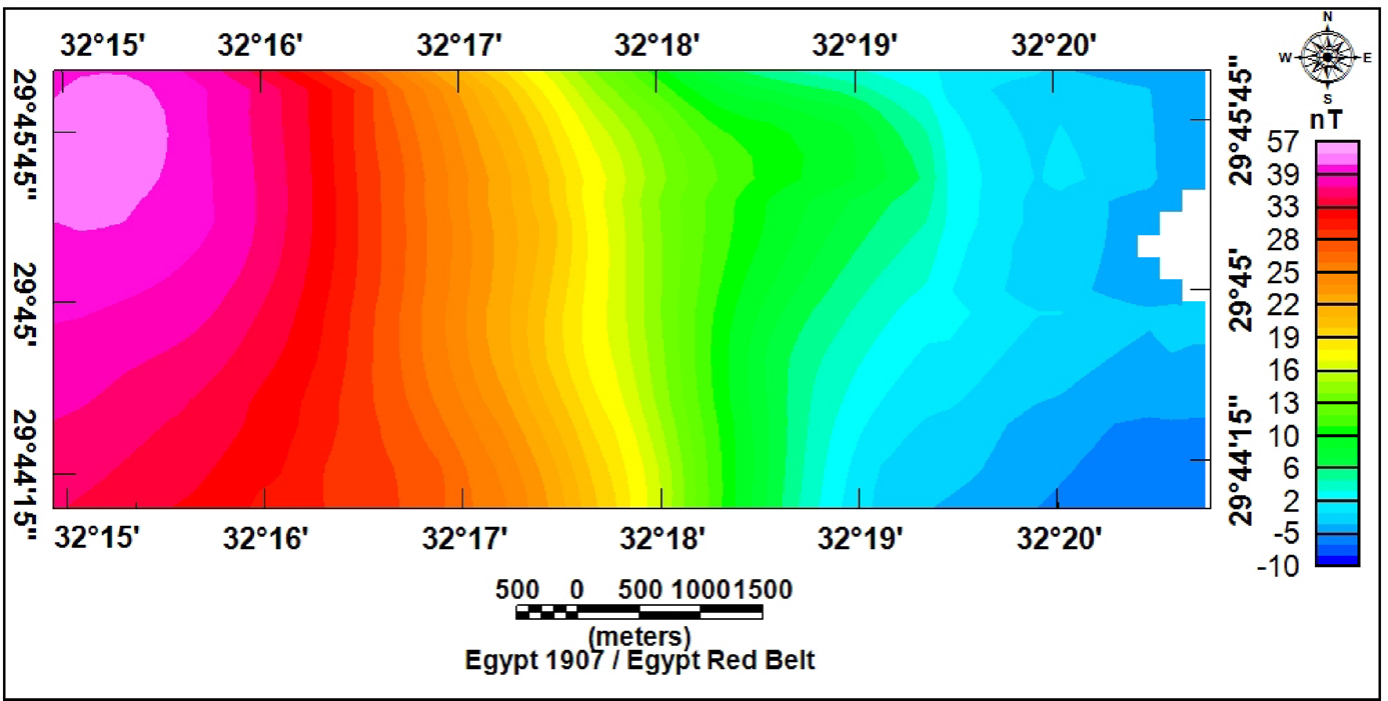
Total intensity magnetic map of the study area.

A total of 183 ground magnetic stations were acquired across the study area (Figs. 1 and 10). The measurements were carried out by using two ENVI-MAG magnetometers, with a sensitivity of 1 nT. Measurement stations were spaced at intervals of 200–500 m. Magnetic data acquisition employed a dual-sensor configuration: one unit served as a mobile magnetometer for field measurements, whereas a second magnetometer acted as a base station to monitor and correct for diurnal variations. Following International Geomagnetic Reference Field (IGRF) subtraction, the resulting TMI anomalies (Fig. 11) were categorized into two primary classes based on their amplitudes.

## Results

### Lineaments and structural analysis

**Fig. 12:**
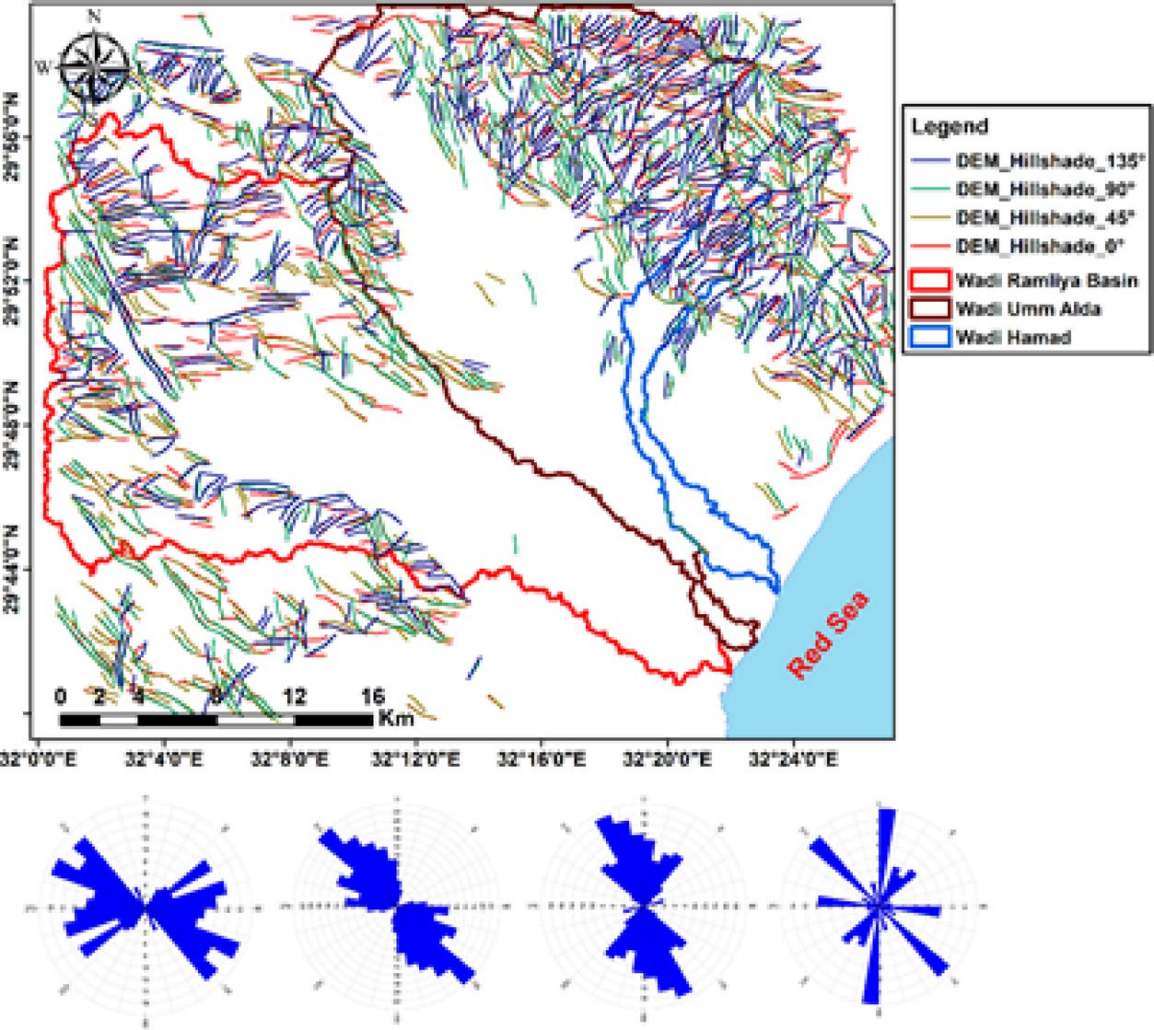
(**a**) Lineament map from shaded-relief imagery at azimuths 0°, 45°, 90°, and 135°; (**b**) Rose diagram (azimuth-frequency) of mapped lineaments.

Lineaments extracted from multi-azimuth hillshade maps (0°, 45°, 90°, 135°) disclose the structural grain of the study area (Fig. 12). The mapped lineaments show dominant orientation in the NW-SW direction, with secondary sets trending in the NE-SW, E–W, and N–S directions. These structural trends correspond to nearby tectonic regimes associated with Red Sea rifting and older basement fabric^17^. The rose diagrams highlight the prevalence of NW–SE systems, which strongly influence the orientation of wadis and drainage pathways, especially in the Ramliya and Umm Alda basins^17^. Overall, the lineament evaluation indicates that drainage and basin morphology are structurally controlled, with fractures and faults providing pathways for surface and subsurface water movement^17^.

**Fig. 13:**
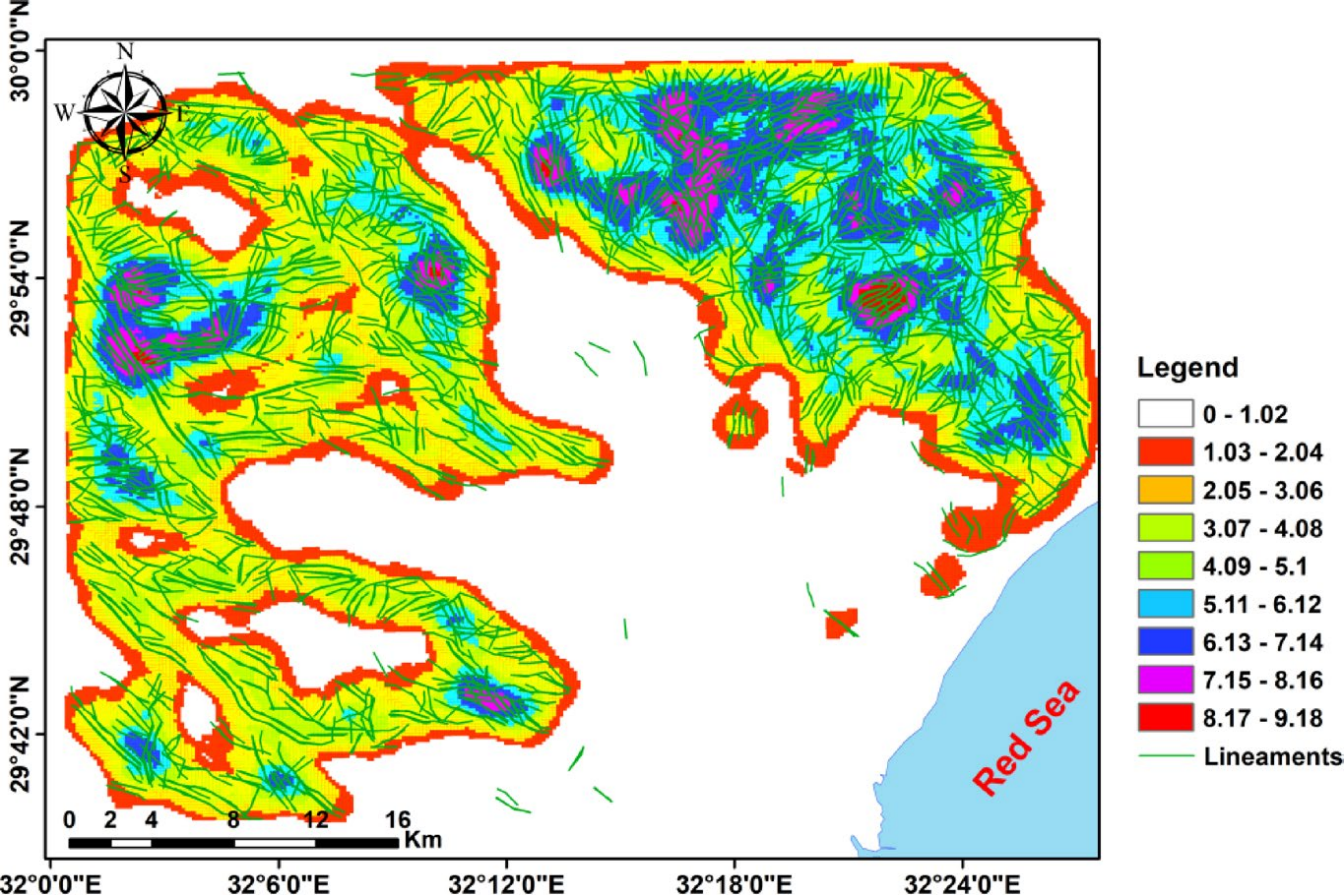
Lineament-density map of the study area.

The lineament density map (Fig. 13) aligns with the DEM and multi-azimuth hillshades: dense lineaments cluster in the rugged highlands and mountain zones (245–1,292 m), whereas the coastal plain and lowlands (17.5–245 m) exhibit low density. Hillshades computed at multiple azimuths consistently highlighted dominant NW–SE and NE–SW structural trends, with subordinate E–W and N–S lineaments, as revealed by the lineament map and rose diagrams. Together, these datasets indicate that ridges and faults influence wadi orientations and drainage pathways toward the Red Sea, suggesting probable zones of enhanced permeability for groundwater recharge. Ridges and faults (Figs. 2 and 13) control wadi directions: fractured/high-relief bedrock routes flow downslope while permeable Pliocene/Quaternary and alluvial deposits at wadi mouths act as focused recharge zones.

### Hydrological processing

**Fig. 14:**
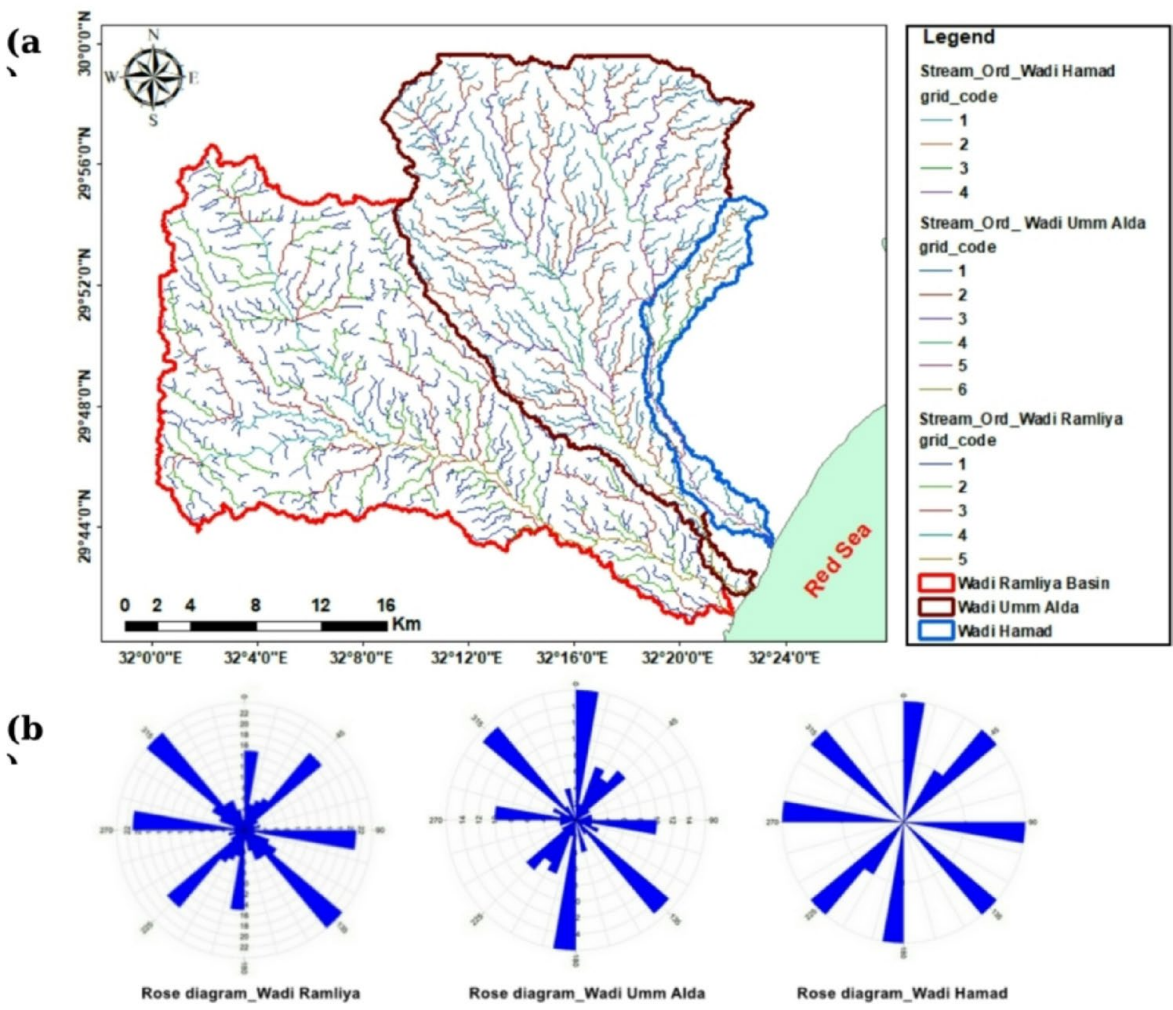
(**a**) Drainage networks of Wadis Ramliya, Umm Alda, and Hamad; (**b**) Rose diagram (azimuth-frequency) of mapped lineaments.

The hydrological processes within the Wadi Ramliya, Wadi Umm Alda, and Wadi Hamad drainage basins are intently inspired via their geomorphological characteristics, including length, shape, gradient, and drainage density^18^. For hydrological evaluation, drainage maps had been extracted from the DEM. The drainage networks have been delineated (Fig. 14), with stream ordering performed according to Strahler^19^. The morphometric metrics applied in this study, along with their computed values, are presented in the table (Table 2), enabling inter-basin comparison and informing interpretations of drainage behavior, structural control, and recharge prioritization.

**Table 2:** Morphometric parameters used for drainage basin Analysis.

Metric	Symbol	Units
Total stream length	*L*_*u*_	km
Number of stream segments	*N*_*u*_	count
Basin area	*A*	km²
Basin perimeter	*P*	km
Mainstream (longest segment) length	*L*_*b*_	km
Basin maximum linear distance (diameter)	*L*_*max*_	km
Drainage density	*D*_*d*_	km · km⁻²
Relief-ratio (texture index)	*R*_*r*_	segments · km⁻¹
Form factor	*F*_*f*_	dimensionless
Min elevation (DEM)	*H*_*min*_	m
Max elevation (DEM)	*H*_*max*_	m
Relief (max − min)	*R*	m
Ruggedness Rn = Dd × relief (relief in m)	*R*_*n*_	(km·km⁻²)×m
Ruggedness Rn (relief in km) — scaled	R*_n_	dimensionless (Dd×relief_km)
Melton’s ruggedness (M)	*M*	m · km⁻¹
Mean bifurcation ratio (mean Rb)	*R*_*b*_	dimensionless

**Table 3:** Morphometric and hydrological parameters of the three study watersheds.

Watershed No.	Wadi Ramliya	Wadi Umm	Wadi Hamad
*L*_*u*_	740.5	645.7	83.69
*N*_*u*_	962	741	88
*A*	452.56	377.37	46.75
*P*	143.33	122.36	66.42
*L*_*b*_	53.22	45.71	28.14
*L*_*max*_	43.41	38.1	21.48
*D*_*d*_	1.64	1.71	1.79
*R*_*r*_	16.57	19.29	31.34
*F*_*f*_	0.1597	0.181	0.059
*H*_*min*_	−5	−5	−5
*H*_*max*_	877	877	877
*R*	882	882	882
*R*_*n*_	1446.48	1509.21	1578.73
R*_n_	1.44	1.51	1.58
*M*	41.46	45.4	128.99
*R*_*b*_	1.46	2	3

**Fig. 15:**
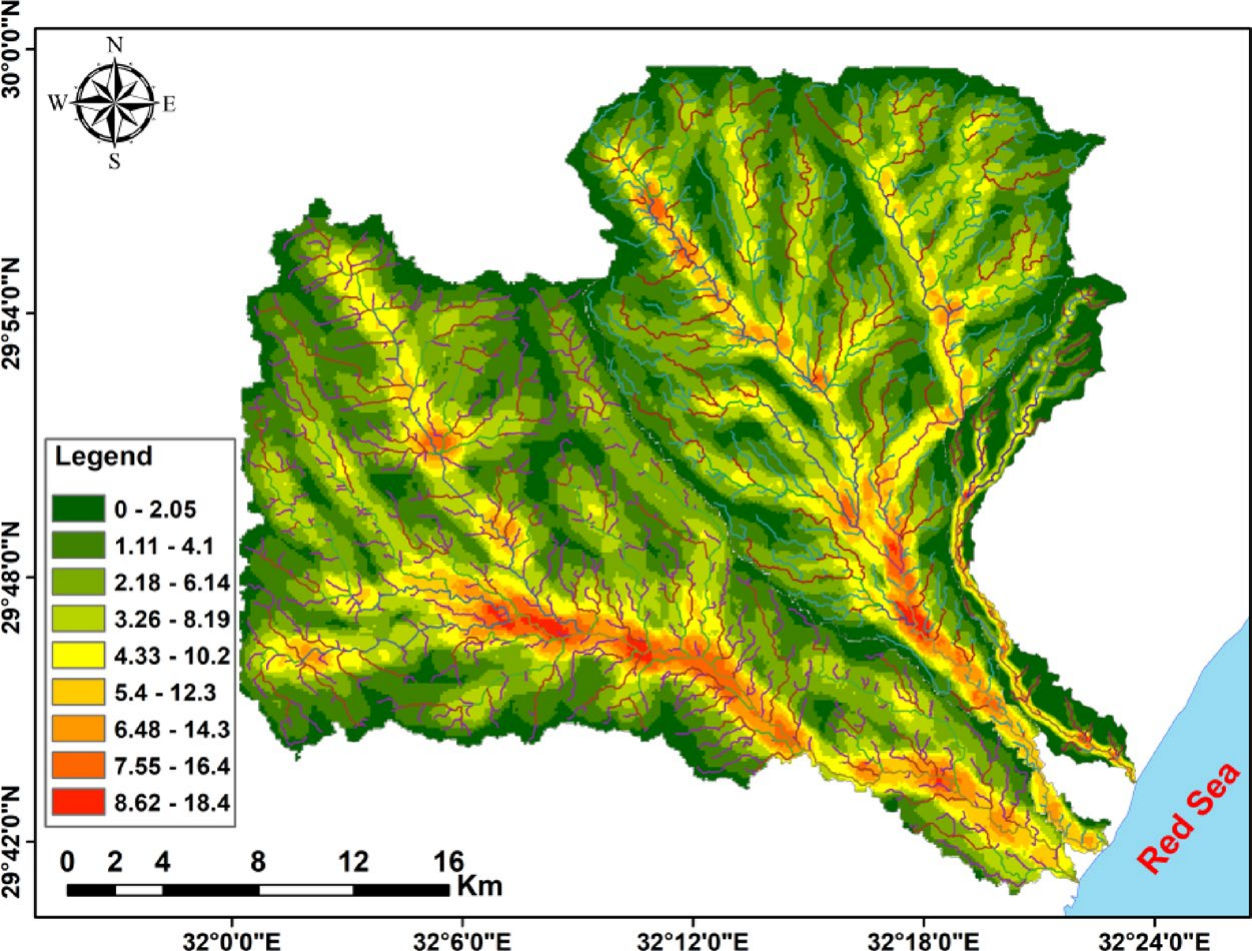
Stream order density map of the Wadis Ramliya, Umm Alda, and Hamad drainage basins.

The integrated analysis, primarily based on DEM topography (Fig. 3), multi-azimuth hillshade lineament extraction (Fig. 12), stream order/density mapping (Figs. 14 and 15), and morphometric metrics summarized in Table 3, was used to characterize the Ramliya, Umm Alda, and Hamad watersheds. The detected spatial patterns of drainage, relief, and lineament density have been coherent with the local stratigraphic framework and with the rift-associated structural grain (dominant NW–SE and NE–SW trends). Together, these datasets have delineated source, transit, and basin zones for surface runoff and groundwater recharge across the study area. DEM analysis revealed four topographic zones that managed the spatial distribution of channel density and drainage orientation (Fig. 15). Multi-azimuth hillshades highlighted the fundamental structural trends: NW–SE and NE–SW lineaments that were dominant within the rugged highlands and mountain bands, and that decreased in density toward the coastal plain. Rose diagrams (Fig. 14b) confirm preferred orientations and linearity of many tributaries, especially in the headwaters. Ridges and faults, therefore, act as watershed divides and structurally guided drainage pathways toward the Red Sea. Morphometric metrics (Table 3) quantified contrasting basin behaviors and aligned with the mapped geology and topography. Wadi Hamad, the smallest basin (A = 46.75 km²), exhibits a disproportionately large total stream length (Lu = 83.69 km) and a high stream count (Nu = 88), producing the highest drainage density (Dd = 1.79 km km⁻²) among the three basins. It also exhibits a high relief ratio (Rr = 31.34), the largest bifurcation ratio (Rb = 3.00), and elevated ruggedness and morphometric indices derived from relief values (e.g., Rn = 1578.73, Rn = 1.58, M = 128.99). These signatures indicate a steep, strongly dissected, structurally constrained headwater system with short concentration times, strong erosive potential, and channel-linearity conditions that have been regular, consistent with the dense lineament clustering observed in the mountainous DEM zones, and with fractures cutting Eocene carbonates and older siliciclastics. Wadi Ramliya, by comparison, had the greatest spatial extent (A = 452.56 km²) and by far the largest total channel network (L_u_ = 740.5 km, N_u_ = 962). Its drainage density is moderate (D_d_= 1.64 km km⁻²). However, simply because its area is so large. Low form factor (Ff = 0.1597), long basin length (L_b_= 53.22 km), and its relief metrics (R_n_ =1446.48, R_n_ =1.44, M = 41.46) all point to a long-transit basin that conveys runoff over greater distances, a configuration that moderates peak discharge and promotes substantial downstream sediment trapping in depositional reaches underlain by Pliocene and Quaternary alluvium. Wadi Umm Alda occupied an intermediate position (A = 377.37 km²; L_u_ = 645.7 km; N_u_ = 741) but displays the highest form factor (F_f_ = 0.181), indicating a more equant basin geometry that tends to disperse runoff and enhance attenuation. Its drainage density (Dd = 1.71 km²⁻²) and relief indicators (Rn = 1509.21, R*n = 1.51, M = 45.4) are likewise intermediate between Ramliya and Hamad, reflecting a balance between upstream structural control and downstream depositional capacity. These morphometric patterns are directly relevant to local lithology and structure, and provide mechanistic explanations for runoff routing and recharge distribution. Eocene carbonate formations (Thebes, Mokattam, Hagul), when exposed or extensively fractured in highlands, fitted out secondary permeability through joints and potential karstic features; wherein dense lineament corridors intersected these carbonates, especially within Hamad and in structurally dissected parts of Ramliya conditions favored deeper fracture mediated percolation and possible spring discharge, consistent with the high stream frequency and inferred subsurface connectivity. Oligocene fluvio-deltaic sandstones (Gabal El-Ahmar, Maadi) and thick Pliocene–Quaternary alluvium, which were represented at lower slopes and wadi mouths, provided high primary porosity and were spatially coincident with low stream order density and low local slope (Fig. 12); these depositional termini therefore represented the principal shallow, focused recharge zones. Tertiary basalts and Neogene volcanics, when present, produced variable responses, characterized by low matrix porosity but significant fracture permeability in jointed intervals, acting locally as rapid conduits aligned with structural lineaments. From the integrated datasets, two complementary recharge regimes were inferred. First, shallow, focused recharge dominated at low-slope wadi mouths and coastal fans (downstream of Ramliya and Umm Alda), where thick Quaternary/Pliocene deposits and lower stream-order density promoted infiltration and aquifer replenishment, making these sites priority goals for controlled aquifer recharge and monitoring. Second, deeper, fracture-mediated recharge became probable when dense lineament corridors intersected permeable Eocene carbonates or Oligocene sandstones, notably in Hamad and in structurally sizable tributaries of Ramliya, where fracture networks supplied vertical and lateral pathways to deeper storage. The morphometric indication, in particular Hamad’s high Dd, Rr, Rb, and relief indices, additionally implied expanded flash-flood susceptibility and excessive sediment yield in headwater sectors; consequently, downstream depositional zones had been recognized as sinks for sediment load, which has direct implications for infiltration efficiency, capacity, aquifer clogging, and the design of sediment management measures in recharge projects.

### Meteorological summary and flash-flood inventory

**Fig. 16:**
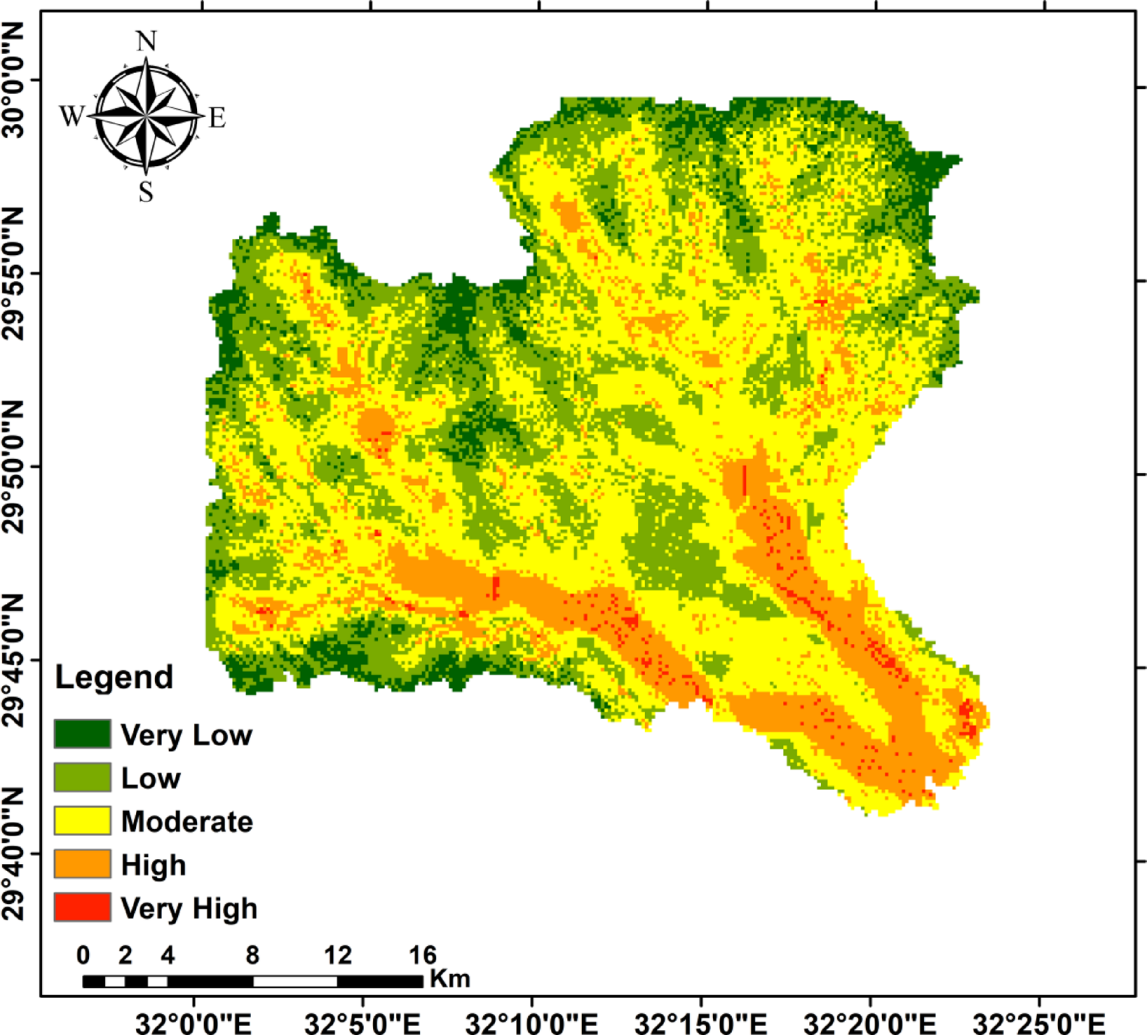
The Flood event map of the three study watersheds.

Analyses of the Suez rain-gauge annual-maximum daily rainfall (1965–2020) and CHIRPS spatial rainfall products confirm that extreme daily rainfall events are episodic but capable of producing rapid runoff in the steeper headwaters. The flood-hazard classification (Fig. 16), produced by combining rainfall intensity with DEM, slope, drainage density, and land-use/cover, highlights very-high to high hazard cells concentrated in the confined fan toes and low-lying coastal plain where wadis discharge (Fig. 16). These hazard zones coincide with the DEM-derived low-slope depositional termini and high drainage-density corridors identified in the morphometric analysis, confirming that the DEM and rainfall data together robustly locate likely flood/recharge sites. Table 1 summarizes the hazard thresholds used (rainfall, drainage density, land-use score, elevation, slope).

### Geoelectrical results

The qualitative interpretation of the VES data resulted in several characteristic resistivity curve types. The frequency and spatial distribution of these species across the study area are summarized in Table 4.

**Table 4:** Summary of the distribution of the different VES Type-Curves in the study Area.

S/*N*	Type Curve	Resistivity Model	VES Number	Frequency	Abundance (%)
1	QH	ρ₁ > ρ₂ > ρ₃ < ρ₄	1, 2, 5, 7, 11, 12, 13, 14, 17, 18, 19, 20, 22, 24, 25, 26, 27, 28	18	64
2	HK	ρ₁ > ρ₂ < ρ₃ > ρ₄	3, 4, 6, 10, 16, 23	6	21
3	QQ	ρ₁ > ρ₂ > ρ₃ > ρ₄	8, 9, 15, 21	1	15
Total				28	100

**Fig. 17:**
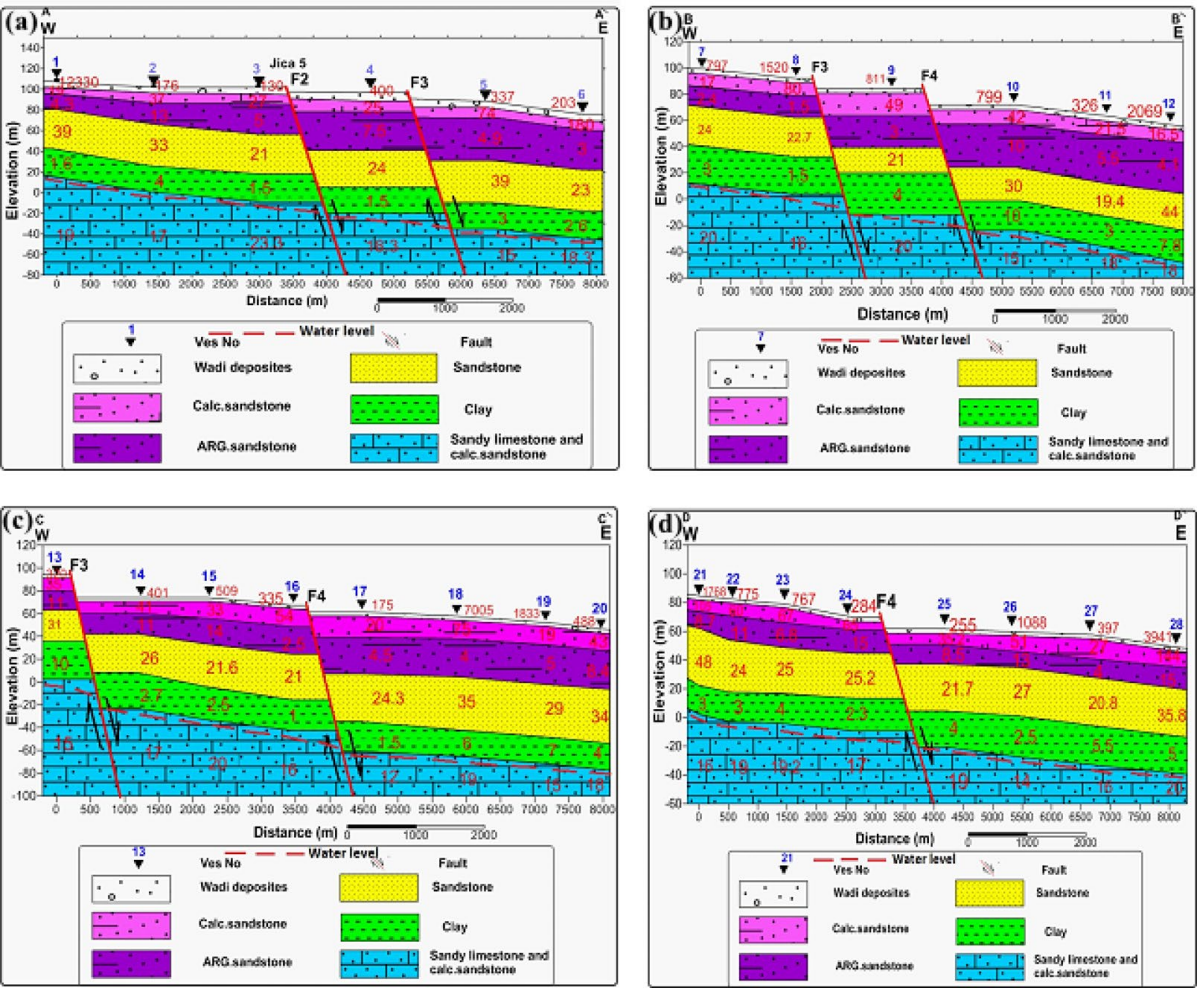
**a-d**. Geoelectrical cross-sections along profiles A–A′, B–B′, C–C′, and D–D.

**Fig. 18:**
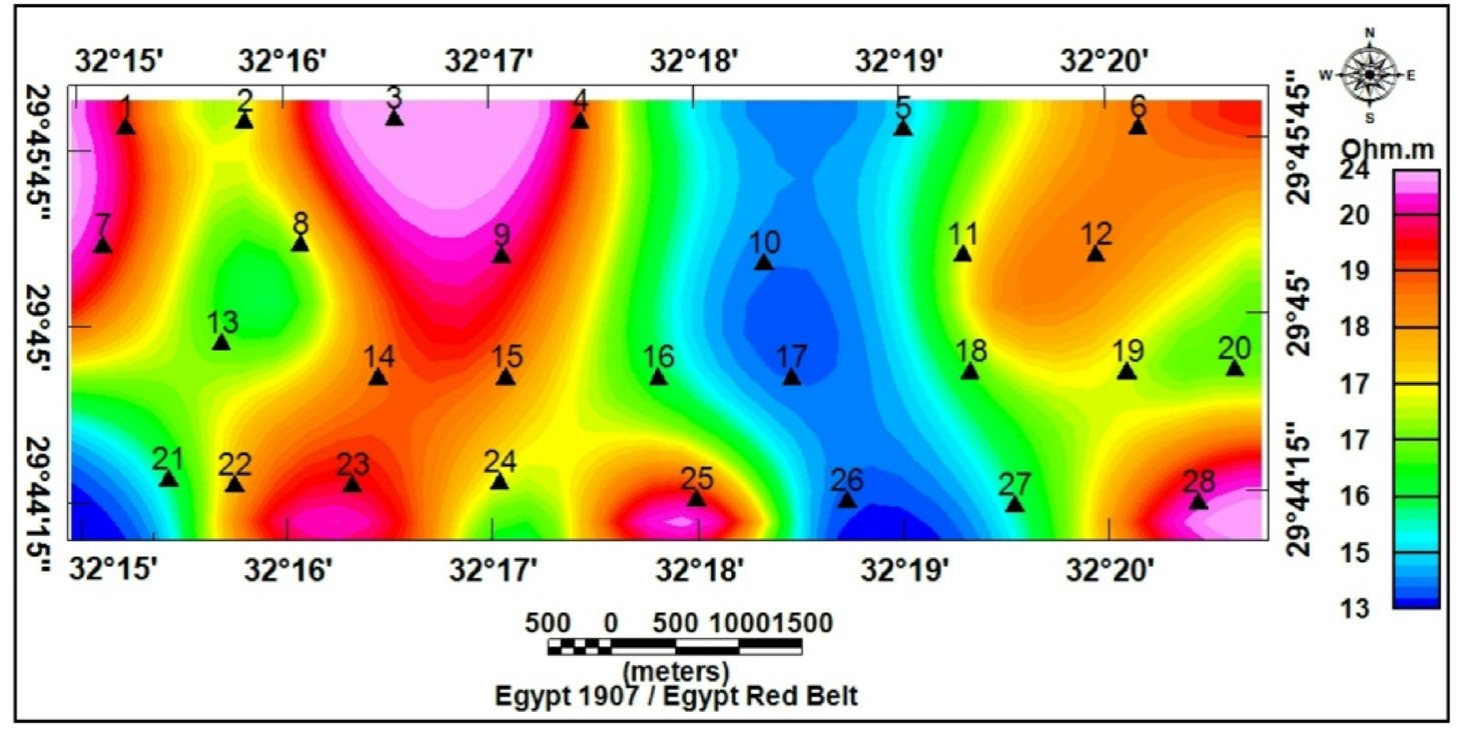
Apparent resistivity map of the sixth layer at the various VES locations.

**Fig. 19:**
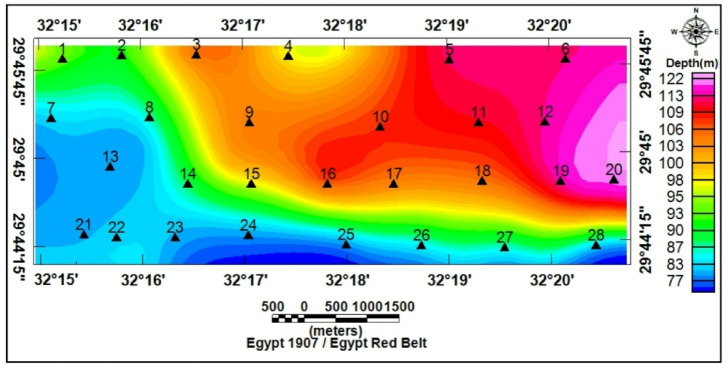
Depth map of the sixth layer at the various VES locations.

1-D inversion of the VES curves produced a consistent six geoelectric layers in the study area. The study area has been subdivided into four geoelectrical cross-sections (A–A′, B–B′, C–C′, and D–D′), from which the six rock layers were consistently resolved (Figs. 17a-d). Interpreted layers involved: (1) Quaternary wadi deposits (gravel/sand) with very high resistivity (130 − 12,330 Ω.m) but thin thickness (0.5–2 m); (2) calcareous sandstone (16.5–180 Ω.m; thickness up to 20 m); (3) argillaceous sandstone/clayey intervals (1.3–15 Ω.m; thickness 4–35 m); (4) sand/sandstone layer (19–48 Ω.m; thickness 17–49 m); (5) clay (1–10 Ω.m; thickness 14–32 m) acting as a potential aquitard; and (6) a deeper calcareous sandstone/sandy limestone aquifer (12–23 Ω.m) occurring at depths of roughly 77–122 m and comprising slightly brackish to fresh water of groundwater of Middle Miocene deposits. For this sixth layer, the true resistivity map (Fig. 18) and the depth map (Fig. 19) had been constructed. The results indicate that the aquifer exhibits relatively uniform resistivity values of 12–23 Ω.m, consistent with fresh groundwater saturation. Depth variations, however, show an overall increase toward the central and northeastern parts of the study area, suggesting that structural and depositional controls influence the aquifer geometry. These six layers provide the basis for compiling depth slices, thickness maps, and interpreted cross-sections, which collectively define the region’s subsurface hydrostratigraphy.

The spatial distribution of the geoelectric layers matched the DEM and stream density patterns: a thin, very resistive layer 1 typically capped depositional fan toes (characterized by low slopes and low stream order termini). Layer 5 (clay) was mapped intermittently as a mid-depth low-resistivity layer; where it was thick and laterally continuous, it characterized an effective barrier to vertical percolation. The deeper layer 6 correlated with mapped Eocene–Miocene carbonate/sandstone successions and regularly lay shallower where lineament intersections and high-relief headwaters suggested structural thinning of the overburden. Thus, geoelectric mapping confirmed that depositional fans (Ramliya and Umm Alda termini) are favorable for shallow, focused recharge, whereas structurally controlled highs (Hamad and lineament nodes) have potential for deeper fracture-aided recharge.

### Petrophysical estimates

**Fig. 20:**
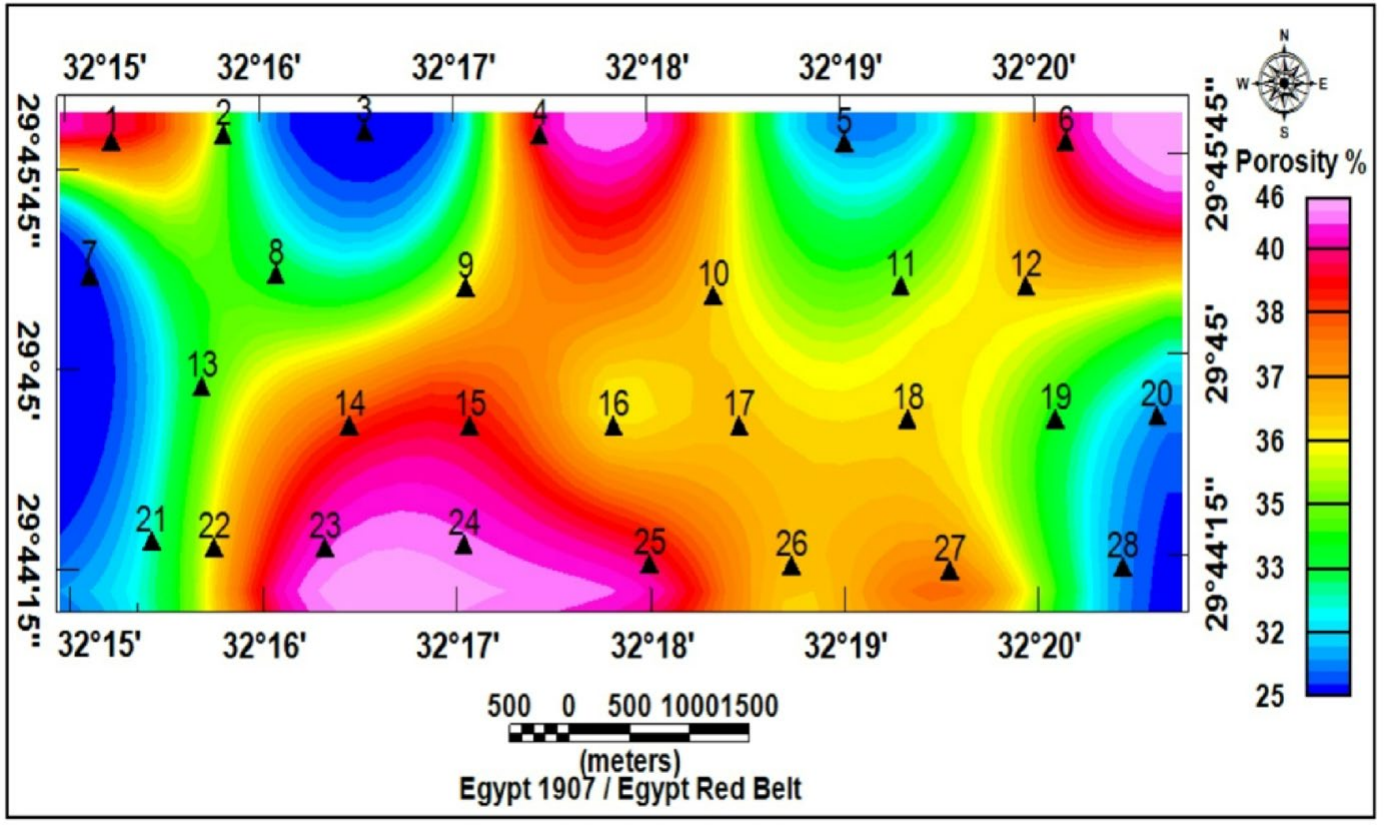
Spatial distribution of porosity (%) in the Middle Miocene aquifer.

Individual VES-based porosity estimates (Table 5) ranged between 38.7% and 48.7%, and displayed a clear spatial trend: porosity increased in the direction of the southern part of the study area and decreased closer to the west (Fig. 20). In this case, *n* = 2 and ρ_t_​ is the true (layer) resistivity; using illustrative average values (F = 6.1, ρ_w_ = 2.85 Ω.m, ρ_t_ = 17.5 Ω.m) produced S_w_=5.6%.

**Table 5:** Porosity values of the middle miocene aquifer (Archie’s equation).

VES.NO	Aquifer resistivity ρ_o_ (Ω.m)	Groundwater resistivity ρ_w_ (Ω.m)	Formation factor F (ρ_o_/ρ_w_)	Porosity φ (%)
1	19	2.85	6.7	38.7
2	17	2.85	6	40.9
3	23.3	2.85	8.2	35
4	18.3	2.85	6.4	39.5
5	15	2.85	5.3	43.6
6	18.3	2.85	6.4	39.5
7	20	2.85	7	37.7
8	16	2.85	5.6	42.2
9	20	2.85	7	37.7
10	15	2.85	5.3	43.6
11	18	2.85	6.3	39.8
12	18	2.85	6.3	39.8
13	15	2.85	5.3	43.6
14	17	2.85	6	40.9
15	20	2.85	7	37.7
16	16	2.85	5.6	42.2
17	12	2.85	4.2	48.7
18	19	2.85	6.7	38.7
19	15	2.85	5.3	43.6
20	18	2.85	6.3	39.8
21	16	2.85	5.6	42.2
22	19	2.85	6.7	38.7
23	19.2	2.85	6.7	38.5
24	17	2.85	6	40.9
25	19	2.85	6.7	38.7
26	14	2.85	4.9	45.1
27	16	2.85	5.6	42.2
28	20	2.85	7	37.7
Average	17.5	2.85	6.1	40.6

This low-resistivity-derived saturation was treated as a first-order indicator of limited effective pore filling (or spatially heterogeneous saturation) and was consequently used only to prioritize field verification, rather than to estimate sustained yields. Hydrochemical data from the JICA-5 calibration borehole (Table 6) showed that the Middle Miocene aquifer water was slightly brackish (TDS = 2,447 mg L⁻¹; EC = 3,330 µS cm⁻¹; pH = 8.2) according to Chebotarev^20^ (Table 7). The moderate aquifer resistivities have been consistent with the interpreted slightly brackish character. Therefore, the deeper (layer-6) resource was considered usable for non-potable applications.

**Table 6:** Statistical summary of groundwater quality data in JICA 5.

Analysis Type	Value	Normal range for drinking water (Ministry of Health Labs)
Mg²⁺ (mg/L)	80.30	150
Ca²⁺ (mg/L)	124	200
Na⁺ (mg/L)	566	
K⁺ (mg/L)	24.50	
SO₄²⁻ (mg/L)	454	250
CO₃²⁻ (mg/L)	48	
HCO₃⁻ (mg/L)	280	
Cl⁻ (mg/L)	887	250
TDS (mg/L)	2447	1500
Electric Conductivity	3330	
Salinity %	2.50	
pH	8.2	6.50–9.20
Al³⁺	-	
Fe³⁺ (mg/L)	-	0.30
Mn²⁺ (mg/L)	0.40	
Zn	-	6.50–9.20
Cu²⁺ (mg/L)	0.01	
Pb²⁺ (mg/L)	0.007	
Cd²⁺ (mg/L)	0.01	

**Table 7:** Classification of Water^20^.

T.D.S. (ppm)	Quality
< 500	Good potable
500–700	Fresh
700–1500	Fairly fresh
1500–2000	Passably fresh
2000–3200	Slightly brackish
3200–4000	Brackish
4000–5000	Definitely brackish
5000–6000	Slightly salty
6000–7000	Salty
7000–10,000	Very salty

### Magnetic data interpretation

**Fig. 21:**
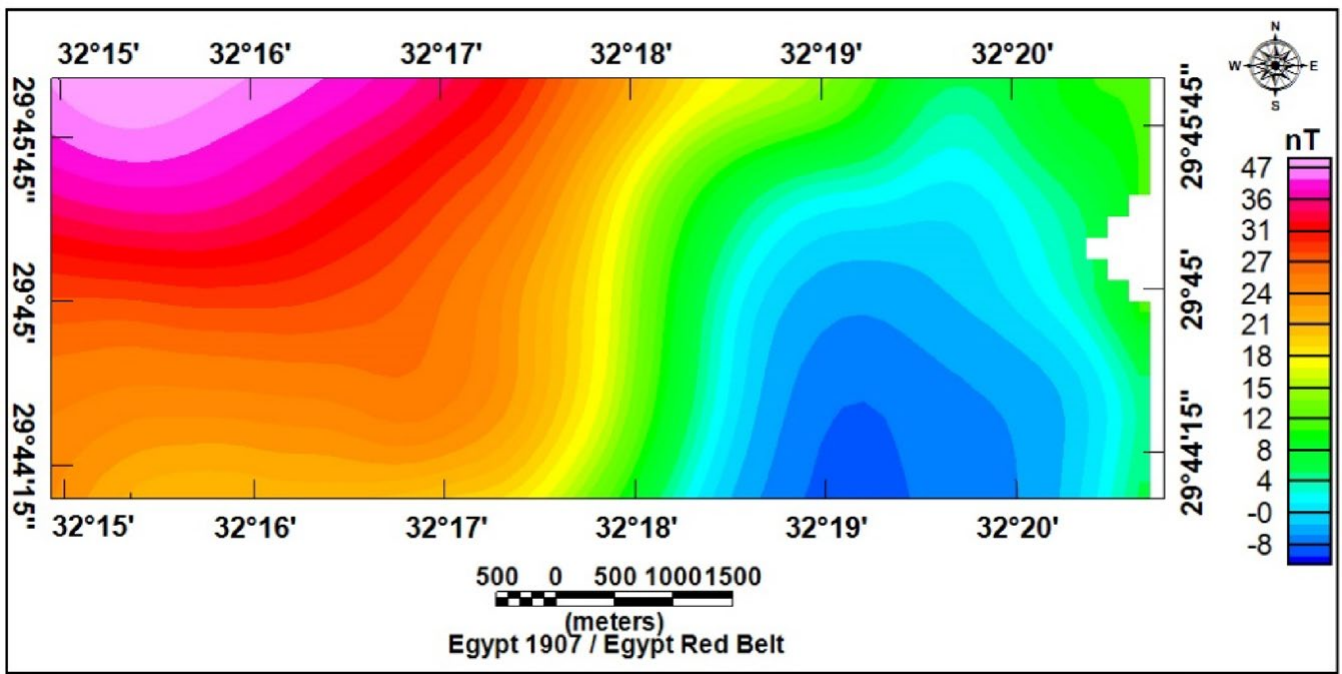
Reduced to the magnetic pole map RTP of the study area.

The RTP magnetic map of the study area (Fig. 21) shows clear spatial variability of magnetic anomalies. Positive anomalies dominate the northwestern part, reflecting uplifted basement rocks of high magnetic susceptibility, with values between (+ 36 and + 47 nT). In contrast, the central and eastern parts exhibit low anomalies (–8 to + 15 nT), indicating thicker sedimentary cover, while the southwestern sector shows intermediate values (+ 15 to + 35 nT).

#### Analytic signal method

The Analytical Signal (AS) is a powerful technique used in the interpretation of magnetic data, primarily for locating the edges of magnetic sources (like faults or contacts) and estimating their depth^21–23^. The analytical signal transforms the complexity of the observed magnetic field into a single, positive-valued quantity. This is crucial because standard magnetic field data (TMI) is dependent on two factors that can complicate interpretation. The first is the direction of magnetization; the signal varies depending on the magnetic properties of the rocks. The second is the Earth’s magnetic field, where the anomaly is skewed (offset from the source) based on the magnetic inclination (the angle at which the Earth’s field dips into the ground). The analytical signal magnitude effectively reduces these directional dependencies, resulting in a magnetic anomaly that is always positioned directly over the edge of the source body, regardless of the magnetization direction.

The analytical signal (∣*A(x*,* y*)∣) is the amplitude of the total gradient vector of the magnetic field (*M*). It is calculated as the square root of the sum of the squares of the derivatives in all three spatial directions (*x*,* y*,* and z*):

5 $$\left| {A(x,y)} \right|\sqrt {{{\left( {\frac{{\partial \:M}}{{\partial \:x}}} \right)}^2} + {{\left( {\frac{{\partial \:M}}{{\partial \:y}}} \right)}^2} + {{\left( {\frac{{\partial \:M}}{{\partial \:z}}} \right)}^2}}$$

Where the horizontal derivatives 𝜕𝑀/𝜕𝑥,𝜕𝑀𝜕𝑦 define the rate of change across the surface, the vertical derivative (𝜕𝑀/𝜕z) defines the rate of change with altitude.

**Fig. 22:**
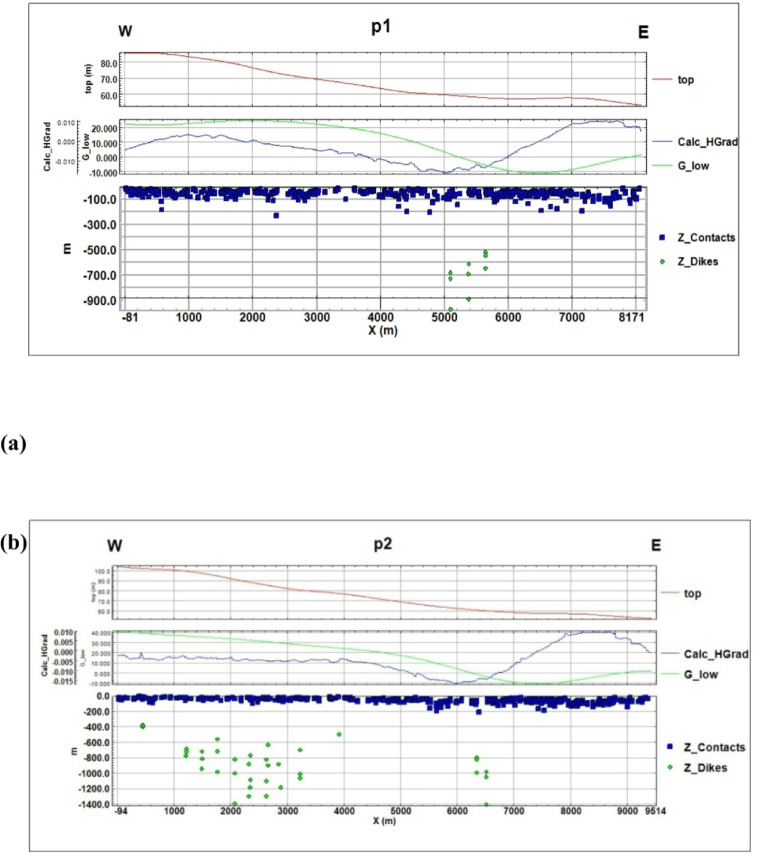
Analytic Signal magnitude at profiles 1 (**a**) and 2 (**b**), showing the distribution of magnetic anomalies used to infer the depth and boundaries of subsurface geological structures.

Analytic signal magnitude processing was applied to the magnetic dataset to estimate the depth to basement along two selected profiles (Figs. 22a and b). The depths derived from the analytic signal on profile 1 range from approximately 500 m to 1,000 m, while profile 2 shows a broader variation, ranging from approximately 400 m to 1,400 m. The shallowest values correspond to basement structural highs, whereas the greatest depths mark structural lows or depocenters. These depth variations outline the major basement morphology beneath the study area and provide important constraints for subsequent structural and basin-architecture interpretations.

#### Delineation of structural elements

**Fig. 23:**
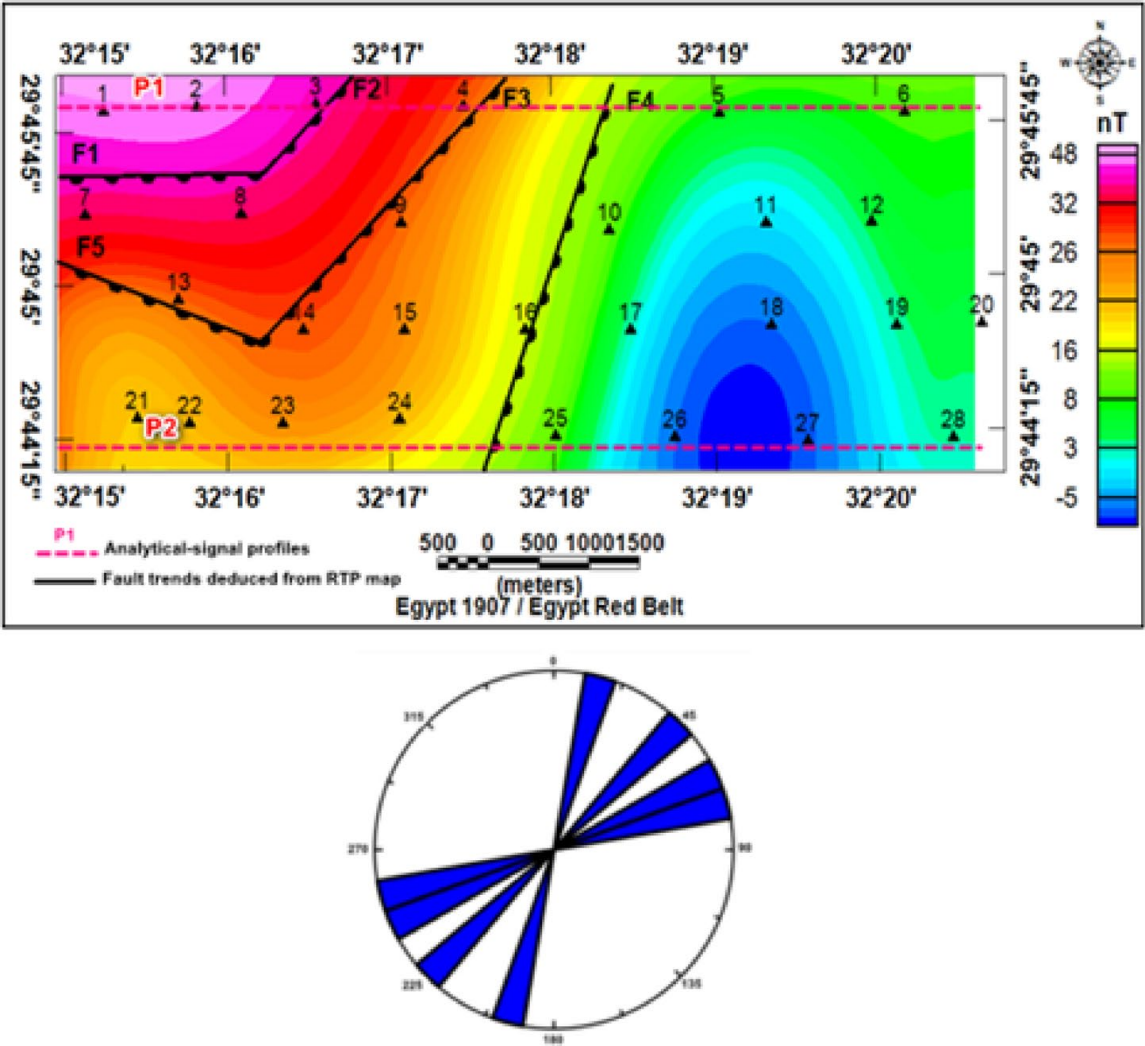
(**a**) Structural Lineaments and Fault Trends Deduced from RTP, (**b**) Rose-Diagram Representation.

Trend and lineament analysis of RTP delineated a dense set of structural elements that dissected the study area (Fig. 23a and b). Faults and lineaments clustered along systematic orientations principally N–S, NW–SE and NE–SW, with subordinate E–W trends and the rose diagram (Fig. 23a and b) emphasized N–S, NW–SE parallel to the Gulf of Suez trend^24^, and NE–SW parallel to the Gulf of Aqaba trend as the dominant fabrics^25^. This tectonic pattern is consistent with the regional Red Sea–Gulf of Suez–Gulf of Aqaba stress field and explains the linear alignment of topographic ridges, the preferred orientations of wadis, and the directional control observed in the stream-order maps^26^. When integrated with the morphometric, DEM, and VES results, the magnetic structural map had direct hydrogeologic implications. Basement highs and faults that correspond to uplifted magnetic sources are associated with areas of thinner sedimentary cover and potentially greater bedrock fracturing. Such structural highs and fault zones are likely to exhibit enhanced vertical and lateral permeability. Conversely, magnetic lows and analytic-signal maxima for depth (i.e., deep depocenters) marked the principal sedimentary basins where Quaternary/Pliocene alluvium and Oligocene sandstones accumulated—these locations coincided with mapped depositional fan toes and low stream-order termini and were identified as the primary shallow recharge and storage zones.

## Discussion

Topographic and structural analysis reveal a strongly partitioned landscape that controls runoff, both in terms of routing and groundwater potential, across the study area. DEM-derived elevation classes— coastal plain (17.5–90 m), intermediate lowlands (90–245 m), highlands (245–577.5 m), and mountainous terrain (577.5–1,292 m)— identify steep northeastern highlands as primary source zones and extensive depositional sinks along the Red Sea margin^1^. Multi-azimuth hillshades highlight dominant NW–SE and NE–SW structural trends and clarify ridge and valley morphology: ridges align with watershed divides and clustered lineament, while interfluves and fan toes correspond to low-slope depositional regions^17^. This topographic structural-framework explain the contrasting hydrologic behaviors between the steep, highly dissected Wadi Hamad and the larger transit/depositional basins of Ramliya and Umm Alda^1^. Morphometric quantified these contrasts: Wadi Hamad, despite being the smallest basin (A = 46.8 km²), shows the highest per-area dissection (D_d_=1.79 km km⁻²), stream frequency (R_r_=31.3) and bifurcation ratio (R_b_=3.0), a signature of short concentration times, repaid peak flows and strong structural control on channels alignment. By contrast, Wadi Ramliya (A = 452.6 km²) and Wadi Umm Alda (A = 377.4 km²) have much greater total channel lengths yet lower drainage density per unit area (Dd = 1.64–1.71 km km⁻²) and higher form factors (relative to Hamad), consistent with longer travel times, runoff attenuation, and enhanced sediment trapping in downstream fans^1^. These quantified morphometric patterns corroborated the DEM and lineament observations, providing an objective basis for separating source, transit, and sink zones for runoff and sediment^1^. The flood hazard assessment combines gauge/CHIRPS datasets and reclassifies them into categorical hazard classes^8^. The results show “very-high” and “high” hazard zones are concentrated at confined alluvial fan toes and coastal plains where wadi discharge occurs. These flood-hazard zones coincide spatially with the DEM-derived low-slope depositional termini and high drainage-density corridors identified in the morphometric analysis, confirming that episodic intense rainfall events focus runoff and sediment into the same depositional sinks that are prime candidates for shallow recharge. Though the same zones are also at greatest risk of rapid sedimentation and infiltration clogging, high sediment loads from flash floods in steep headwaters (especially Wadi Hamad) are likely to reduce infiltration efficiency in fan toes unless sediment control measures are implemented. Geoelectrical imaging (28 VES) resolved a consistent six-layer hydrostratigraphy and mapped the aquifer geometry with good lateral continuity^15^. Shallow, highly resistive Quaternary wadi deposits (layer-1) commonly capped coarser fan toes; beneath these sand and sandstone lenses (layer-4; 19–48 Ω.m; thickness 17–49 m) represent the principal shallow-intermediate aquifer targets for managed recharge and short-term abstraction. Intermittent clay-rich intervals (layer-5; 1–10 Ω.m; thickness 14–32 m) act as potential aquitards and pose clogging risks for the infiltration scheme. The deeper, regionally important Middle Miocene calcareous sandstone and sandy limestone aquifer (layer-6) was imaged at depths of 77–122 m with resistivities between 12 and 23 Ω.m ^6^. Layer 6 was generally shallower above basement highs and along lineament corridors and deeper within magnetic depocenters, a spatial pattern that increases confidence in the interpreted aquifer geometry. Petrophysical transforms and formation-water data (ρ_w_ = 2.85 Ω.m from JICA-5) further refine the picture: applying Archie’s law (a = 1, m = 2) yields formation factors of 4.2–8.2 and porosity estimates of 35–49% (mean 40.6%), indicating substantial storage potential. Resistivity-derived saturation estimates (representative averages) produce S_w_ = 5.6%, but this value is flagged as sensitive because it is highly sensitive to Archie parameters and pore connectivity. Hydrochemistry from JICA-5 (TDS = 2,447 mg L⁻¹; EC = 3,330 µS cm⁻¹; pH = 8.2) classifies the deeper Middle Miocene aquifer as slightly brackish, limiting direct potable use without treatment or blending, but suitable for selected non-potable applications (e.g., irrigation, industrial uses). Independent magnetic surveys reinforce the subsurface interpretation: Total magnetic and RTP maps high-amplitude anomalies and shallower analytical-signal depths over uplifted basement blocks (interpreted as thin cover zones)^21–23^, whereas low-amplitude anomalies and deeper analytical-signal maxima mark sedimentary fill-depocenters. Analytical-signal depths estimated to the basement range from 400 to 1,400 m along different transects, revealing a complex basement morphology that controls the accommodation space for Neogene–Quaternary deposits. These magnetic fabrics corresponded with tectonic lineaments mapped at the surface, confirming that both aquifer depth and sediment distribution were structurally controlled by Red Sea–Gulf of Suez–Aqaba rift trends^2,3^. The correlation between magnetic depocenters and DEM-defined basin floors supported the interpretation that structural lows collected the thickest alluvium and were therefore the most likely site for substantial shallow aquifer development.

Although some interpretation limitations, including magnetic non-uniqueness, the limited lateral resolution of 1-D VES, and the availability of a rain gauge located 40 km from the study area, the integrated methodology produced robust, target-scale outcomes by calibrating land magnetic and geoelectric interpretations with borehole logs and by using CHIRPS to supplement sparse gauge data. The multi-dataset approach effectively discriminated source transit sink zones and produced prioritized, field-testable targets for recharge and reconnaissance drilling. The mapped fan toes and lineament intersections represent the highest near-term potential for augmenting local groundwater, but realization of that potential requires concurrent sediment management, rigorous hydrogeological testing, and a sustained monitoring program to ensure long-term recharge effectiveness and aquifer protection.

## Conclusions

This study successfully employed an integrated hydrogeophysical and morphometric approach to characterize the subsurface structure, hydro-stratigraphy, and recharge potential across the Wadi Ramliya, Wadi Umm Alda, and Wadi Hamad basins, establishing a scientific foundation for groundwater management in this arid region. The integrated DEM, geoelectrical, and magnetic study verified that the hydrogeology of the Ramliya, Umm Alda, and Hamad basins was structurally controlled. DEM and hillshades showing NW–SE and NE–SW lineaments that served as watershed divides and guided drainage pathways, whereas magnetic highs and lows defined basement blocks and depocenters controlled sediment thickness and aquifer geometry. Flood-hazard mapping (Suez gauge/CHIRPS/reclassified DEM, slope, drainage density, land-use/cover, and elevation) revealed very high to high hazard zones concentrated at confined fan toes and the coastal plain. These zones coincide with the best shallow-recharge candidates but also face a high risk of sedimentation/clogging. VES imaging resolved six geoelectric layers and pointed out a local Middle Miocene aquifer (layer-6) at 77–122 m (ρ = 12–23 Ω.m). Archie’s transforms yielded porosities of 35–49% (mean ≈ 40.6%), and JICA-5 chemistry indicated slightly brackish water (TDS = 2,447 mg·L⁻¹). The recommendations and future research can include hydrogeological modeling, such as developing a 3D groundwater flow model incorporating structural boundaries (faults derived from the Analytical Signal) and precise thickness estimates (derived from VES) to predict the response of the Middle Miocene aquifer to sustained pumping. Refined petrophysics, conducting advanced borehole logging (e.g., nuclear magnetic resonance) on future reconnaissance wells to obtain in situ measurements of effective porosity and hydraulic conductivity, which would reduce the inherent uncertainty associated with surface-based resistivity and Archie’s Law transforms.

In summary, the multi-dataset approach effectively discriminated source transit sink zones and produced prioritized, field-testable targets for recharge and reconnaissance drilling. The mapped fan toes and lineament intersections represent the highest near-term potential to augment local groundwater, but realizing that potential requires concurrent sediment management, rigorous hydrogeological testing, and a sustained monitoring program to ensure long-term recharge effectiveness and aquifer protection.

## Data Availability

The data that support the findings of this study are available from the corresponding author upon reasonable request.
